# Supplementary material for: Cell Type Impacts Accessibility of mRNA to Silencing by RNA Interference
Source: Mol Ther Nucleic Acids. 2020 Jun 12;21:384–93. doi: 10.1016/j.omtn.2020.06.006 (PMC7340969; doi:10.1016/j.omtn.2020.06.006)
Supplement: Document S1. Figures S1 and S2 and Tables S1–S3 [file mmc1.pdf]

OMTN, Volume 21

Supplemental Information

Cell Type Impacts Accessibility of mRNA

to Silencing by RNA Interference

Chantal M. Ferguson, Dimas Echeverria, Matthew Hassler, Socheata Ly, and Anastasia Khvorova

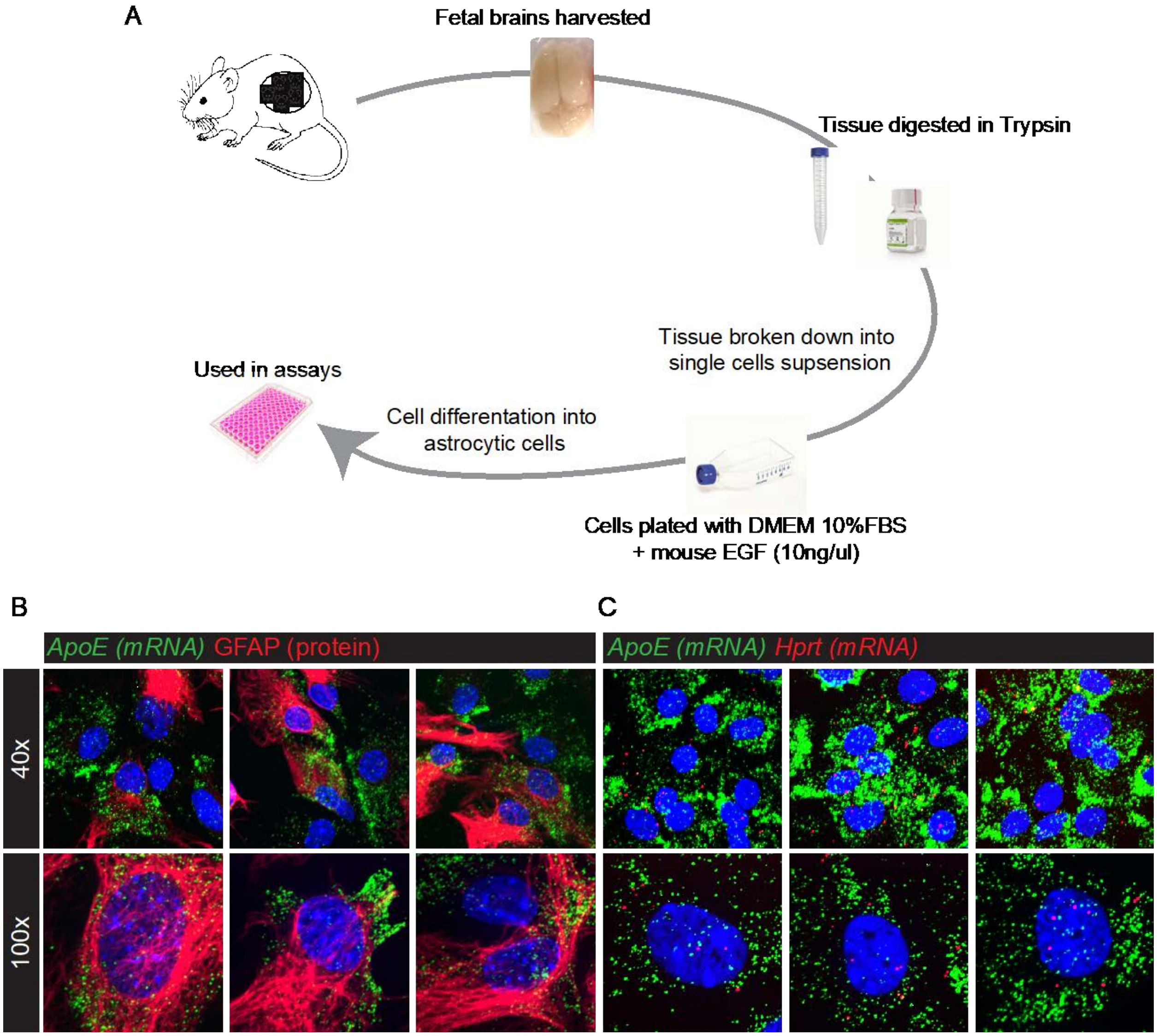

Supplementary Figure 1: Preparation of primary mouse astrocytes. (A) Workflow for harvest and preparation. (B) Primary mouse astrocytes co-stained for *ApoE* mRNA (green) (RNAscope) and GFAP protein (red) (IF) at 40x and 100x magnification confirming astrocyte lineage. (C) Primary mouse astrocytes co-stained for *ApoE* (green) and *HPRT* (red) mRNA (RNAscope) at 40x and 100x magnification.

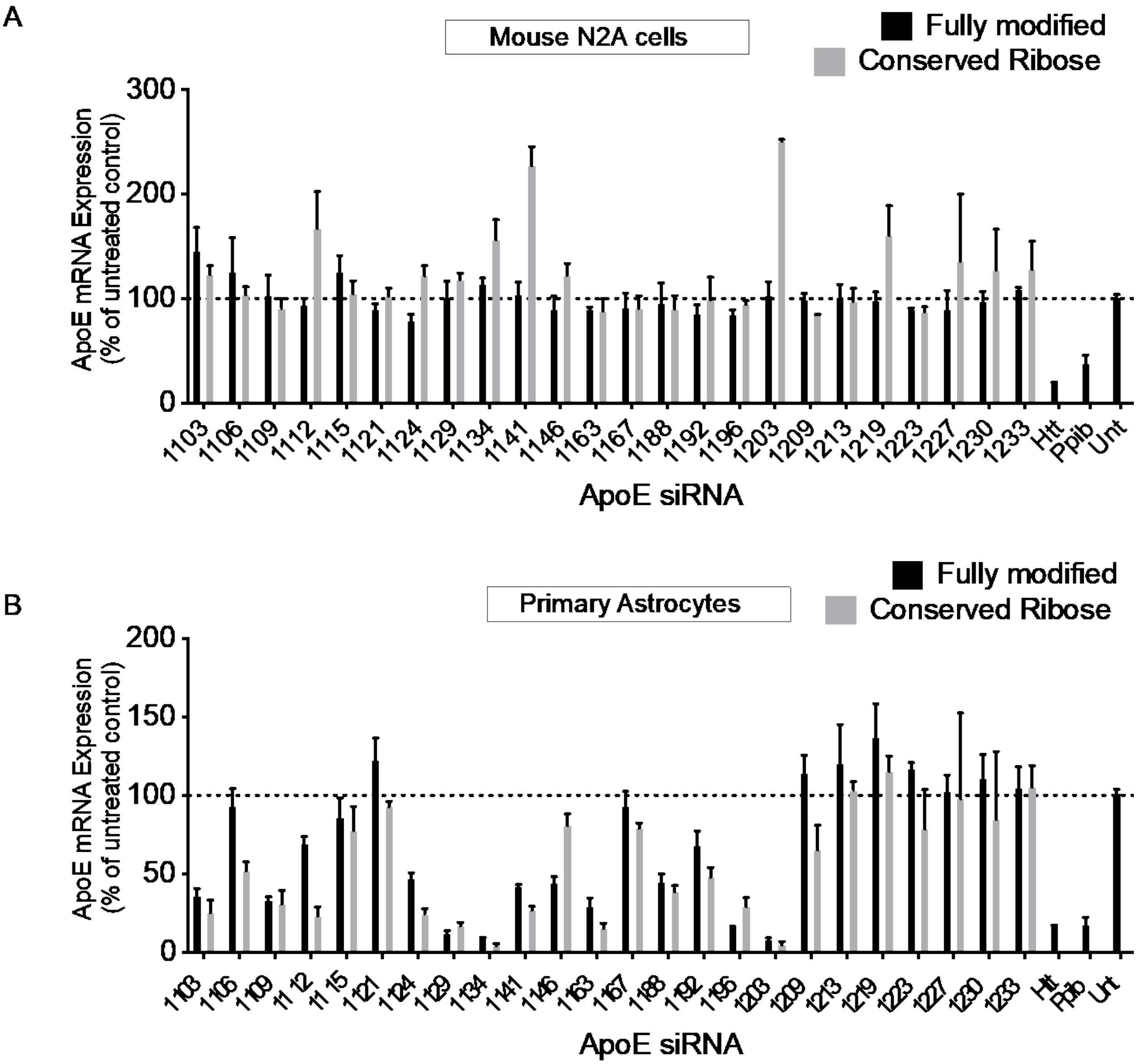

Supplementary Figure 2: Conservation of 2'OH (Ribose) does not impact RNAi efficacy. (A). Screen using fully modified (black) and partially modified (grey) siRNAs targeting *ApoE* in N2A cells. (B) Screen using fully modified (black) and partially modified (grey) siRNAs targeting *ApoE* in mouse primary astrocytes. Dose: 1.5 µM; 72 -hour timepoint; mRNA quantified using QuantiGene.

Table 1: siRNA sequences and chemical modifications

Name	Gene	Accession Number	Anti-sense strand	Sense strand
Apoe_156	Mouse ApoE	NM_009696	P(mU)#(fC)#(mA)(fA)(mU)(fU)(mG)(fU)(mG)(fA)(mU)(fU)(mG)#(fG)#(mC)#(fC)#(mA)#(fG)#(mU)#(fC)	(fG)#(mC)#(fC)(mA)(fA)(mU)(fC)(mA)(fC)(mA)(fA)(mU)(fU)#(mG)#(fA)-TegChol
Apoe_416	Mouse ApoE	NM_009696	P(mU)#(fU)#(mU)(fU)(mG)(fU)(mA)(fA)(mG)(fC)(mC)(fU)(mU)#(fU)#(mA)#(fC)#(mU)#(fU)#(mC)#(fC)	(fU)#(mA)#(fA)(mA)(fG)(mG)(fC)(mU)(fU)(mA)(fC)(mA)(fA)#(mA)#(fA)-TegChol
Apoe_636	Mouse ApoE	NM_009696	P(mU)#(fU)#(mC)(fA)(mA)(fG)(mC)(fG)(mC)(fU)(mU)(fG)(mC)#(fG)#(mC)#(fA)#(mU)#(fC)#(mU)#(fU)	(fG)#(mC)#(fG)(mC)(fA)(mA)(fG)(mC)(fG)(mC)(fU)(mU)(fG)#(mA)#(fA)-TegChol
Apoe_1004	Mouse ApoE	NM_009696	P(mU)#(fG)#(mU)(fC)(mU)(fU)(mC)(fC)(mA)(fC)(mU)(fA)(mU)#(fU)#(mG)#(fG)#(mC)#(fU)#(mC)#(fG)	(fC)#(mA)#(fA)(mU)(fA)(mG)(fU)(mG)(fG)(mA)(fA)(mG)(fA)#(mC)#(fA)-TegChol
Apoe_1032	Mouse ApoE	NM_009696	P(mU)#(fC)#(mC)(fA)(mU)(fC)(mA)(fG)(mG)(fU)(mU)(fU)(mG)#(fC)#(mC)#(fC)#(mA)#(fC)#(mU)#(fG)	(fG)#(mG)#(fC)(mA)(fA)(mA)(fC)(mC)(fU)(mG)(fA)(mU)(fG)#(mG)#(fA)-TegChol
Apoe_1103	Mouse ApoE	NM_009696	P(mU)#(fG)#(mG)(fA)(mU)(fA)(mC)(fU)(mC)(fA)(mU)(fU)(mG)#(fA)#(mU)#(fU)#(mC)#(fU)#(mC)#(fC)	(fA)#(mU)#(fC)(mA)(fA)(mU)(fG)(mA)(fG)(mU)(fA)(mU)(fC)#(mC)#(fA)-TegChol
Apoe_1106	Mouse ApoE	NM_009696	P(mU)#(fG)#(mA)(fA)(mG)(fG)(mA)(fU)(mA)(fC)(mU)(fC)(mA)#(fU)#(mU)#(fG)#(mA)#(fU)#(mU)#(fC)	(fA)#(mA)#(fU)(mG)(fA)(mG)(fU)(mA)(fU)(mC)(fC)(mU)(fU)#(mC)#(fA)-TegChol
Apoe_1109	Mouse ApoE	NM_009696	P(mU)#(fG)#(mG)(fA)(mG)(fA)(mA)(fG)(mG)(fA)(mU)(fA)(mC)#(fU)#(mC)#(fA)#(mU)#(fU)#(mG)#(fA)	(fG)#(mA)#(fG)(mU)(fA)(mU)(fC)(mC)(fU)(mU)(fC)(mU)(fC)#(mC)#(fA)-TegChol
Apoe_1112	Mouse ApoE	NM_009696	P(mU)#(fA)#(mC)(fA)(mG)(fG)(mA)(fG)(mA)(fA)(mG)(fG)(mA)#(fU)#(mA)#(fC)#(mU)#(fC)#(mA)#(fU)	(fU)#(mA)#(fU)(mC)(fC)(mU)(fU)(mC)(fU)(mC)(fC)(mU)(fG)#(mU)#(fA)-TegChol
Apoe_1115	Mouse ApoE	NM_009696	P(mU)#(fA)#(mG)(fG)(mA)(fC)(mA)(fG)(mG)(fA)(mG)(fA)(mA)#(fG)#(mG)#(fA)#(mU)#(fA)#(mC)#(fU)	(fC)#(mC)#(fU)(mU)(fC)(mU)(fC)(mC)(fU)(mG)(fU)(mC)(fC)#(mU)#(fA)-TegChol
Apoe_1119	Mouse ApoE	NM_009696	P(mU)#(fU)#(mU)(fG)(mC)(fA)(mG)(fG)(mA)(fC)(mA)(fG)(mG)#(fA)#(mG)#(fA)#(mA)#(fG)#(mG)#(fA)	(fC)#(mU)#(fC)(mC)(fU)(mG)(fU)(mC)(fC)(mU)(fG)(mC)(fA)#(mA)#(fA)-TegChol
Apoe_1121	Mouse ApoE	NM_009696	P(mU)#(fU)#(mG)(fU)(mU)(fG)(mC)(fA)(mG)(fG)(mA)(fC)(mA)#(fG)#(mG)#(fA)#(mG)#(fA)#(mA)#(fG)	(fC)#(mC)#(fU)(mG)(fU)(mC)(fC)(mU)(fG)(mC)(fA)(mA)(fC)#(mA)#(fA)-TegChol
Apoe_1123	Mouse ApoE	NM_009696	P(mU)#(fG)#(mU)(fU)(mG)(fU)(mU)(fG)(mC)(fA)(mG)(fG)(mA)#(fC)#(mA)#(fG)#(mG)#(fA)#(mG)#(fA)	(fU)#(mG)#(fU)(mC)(fC)(mU)(fG)(mC)(fA)(mA)(fC)(mA)(fA)#(mC)#(fA)-TegChol
Apoe_1124	Mouse ApoE	NM_009696	P(mU)#(fU)#(mG)(fU)(mU)(fG)(mU)(fU)(mG)(fC)(mA)(fG)(mG)#(fA)#(mC)#(fA)#(mG)#(fG)#(mA)#(fG)	(fG)#(mU)#(fC)(mC)(fU)(mG)(fC)(mA)(fA)(mC)(fA)(mA)(fC)#(mA)#(fA)-TegChol
Apoe_1126	Mouse ApoE	NM_009696	P(mU)#(fG)#(mA)(fU)(mG)(fU)(mU)(fG)(mU)(fU)(mG)(fC)(mA)#(fG)#(mG)#(fA)#(mC)#(fA)#(mG)#(fG)	(fC)#(mC)#(fU)(mG)(fC)(mA)(fA)(mC)(fA)(mA)(fC)(mA)(fU)#(mC)#(fA)-TegChol
Apoe_1129	Mouse ApoE	NM_009696	P(mU)#(fA)#(mU)(fG)(mG)(fA)(mU)(fG)(mU)(fU)(mG)(fU)(mU)#(fG)#(mC)#(fA)#(mG)#(fG)#(mA)#(fC)	(fG)#(mC)#(fA)(mA)(fC)(mA)(fA)(mC)(fA)(mU)(fC)(mC)(fA)#(mU)#(fA)-TegChol
Apoe_1134	Mouse ApoE	NM_009696	P(mU)#(fU)#(mG)(fG)(mA)(fU)(mA)(fU)(mG)(fG)(mA)(fU)(mG)#(fU)#(mU)#(fG)#(mU)#(fU)#(mG)#(fC)	(fA)#(mA)#(fC)(mA)(fU)(mC)(fC)(mA)(fU)(mA)(fU)(mC)(fC)#(mA)#(fA)-TegChol
Apoe_1141	Mouse ApoE	NM_009696	P(mU)#(fA)#(mC)(fC)(mU)(fG)(mG)(fC)(mU)(fG)(mG)(fA)(mU)#(fA)#(mU)#(fG)#(mG)#(fA)#(mU)#(fG)	(fA)#(mU)#(fA)(mU)(fC)(mC)(fA)(mG)(fC)(mC)(fA)(mG)(fG)#(mU)#(fA)-TegChol
Apoe_1146	Mouse ApoE	NM_009696	P(mU)#(fG)#(mG)(fG)(mC)(fC)(mA)(fC)(mC)(fU)(mG)(fG)(mC)#(fU)#(mG)#(fG)#(mA)#(fU)#(mA)#(fU)	(fC)#(mA)#(fG)(mC)(fC)(mA)(fG)(mG)(fU)(mG)(fG)(mC)(fC)#(mC)#(fA)-TegChol
Apoe_1163	Mouse ApoE	NM_009696	P(mU)#(fG)#(mA)(fG)(mA)(fG)(mG)(fU)(mG)(fC)(mU)(fU)(mG)#(fA)#(mG)#(fA)#(mC)#(fA)#(mG)#(fG)	(fC)#(mU)#(fC)(mA)(fA)(mG)(fC)(mA)(fC)(mC)(fU)(mC)(fU)#(mC)#(fA)-TegChol
Apoe_1167	Mouse ApoE	NM_009696	P(mU)#(fG)#(mC)(fC)(mA)(fG)(mA)(fG)(mA)(fG)(mG)(fU)(mG)#(fC)#(mU)#(fU)#(mG)#(fA)#(mG)#(fA)	(fA)#(mG)#(fC)(mA)(fC)(mC)(fU)(mC)(fU)(mC)(fU)(mG)(fG)#(mC)#(fA)-TegChol

Apoe_1188	Mouse ApoE	NM_009696	P(mU)#(fU)#(mU)(fA)(mA)(fG)(mC)(fA)(mA)(fG)(mG)(fG)(mC)#(fC)#(mA)#(fC)#(mC)#(fA)#(mG)#(fA)	(fU)#(mG)#(fG)(mC)(fC)(mC)(fU)(mU)(fG)(mC)(fU)(mU)(fA)#(mA)#(fA)-TegChol
Apoe_1191	Mouse ApoE	NM_009696	P(mU)#(fU)#(mU)(fA)(mU)(fU)(mA)(fA)(mG)(fC)(mA)(fA)(mG)#(fG)#(mG)#(fC)#(mC)#(fA)#(mC)#(fC)	(fC)#(mC)#(fC)(mU)(fU)(mG)(fC)(mU)(fU)(mA)(fA)(mU)(fA)#(mA)#(fA)-TegChol
Apoe_1192	Mouse ApoE	NM_009696	P(mU)#(fU)#(mU)(fU)(mA)(fU)(mU)(fA)(mA)(fG)(mC)(fA)(mA)#(fG)#(mG)#(fG)#(mC)#(fC)#(mA)#(fC)	(fC)#(mC)#(fU)(mU)(fG)(mC)(fU)(mU)(fA)(mA)(fU)(mA)(fA)#(mA)#(fA)-TegChol
Apoe_1196	Mouse ApoE	NM_009696	P(mU)#(fA)#(mA)(fU)(mC)(fU)(mU)(fU)(mA)(fU)(mU)(fA)(mA)#(fG)#(mC)#(fA)#(mA)#(fG)#(mG)#(fG)	(fG)#(mC)#(fU)(mU)(fA)(mA)(fU)(mA)(fA)(mA)(fG)(mA)(fU)#(mU)#(fA)-TegChol
Apoe_1203	Mouse ApoE	NM_009696	P(mU)#(fC)#(mU)(fC)(mG)(fG)(mA)(fG)(mA)(fA)(mU)(fC)(mU)#(fU)#(mU)#(fA)#(mU)#(fU)#(mA)#(fA)	(fA)#(mA)#(fA)(mG)(fA)(mU)(fU)(mC)(fU)(mC)(fC)(mG)(fA)#(mG)#(fA)-TegChol
Apoe_1209	Mouse ApoE	NM_009696	P(mU)#(fA)#(mA)(fU)(mG)(fU)(mG)(fC)(mU)(fC)(mG)(fG)(mA)#(fG)#(mA)#(fA)#(mU)#(fC)#(mU)#(fU)	(fU)#(mC)#(fU)(mC)(fC)(mG)(fA)(mG)(fC)(mA)(fC)(mA)(fU)#(mU)#(fA)-TegChol
Apoe_1213	Mouse ApoE	NM_009696	P(mU)#(fU)#(mC)(fA)(mG)(fA)(mA)(fU)(mG)(fU)(mG)(fC)(mU)#(fC)#(mG)#(fG)#(mA)#(fG)#(mA)#(fA)	(fC)#(mG)#(fA)(mG)(fC)(mA)(fC)(mA)(fU)(mU)(fC)(mU)(fG)#(mA)#(fA)-TegChol
Apoe_1213	Mouse ApoE	NM_009696	P(mU)#(fU)#(mC)(fA)(mG)(fA)(mA)(fU)(mG)(fU)(mG)(fC)(mU)#(fC)#(mG)#(fG)#(mA)#(fG)#(mA)#(fA)	(fC)#(mG)#(fA)(mG)(fC)(mA)(fC)(mA)(fU)(mU)(fC)(mU)(fG)#(mA)#(fA)-TegChol
Apoe_1217	Mouse ApoE	NM_009696	P(mU)#(fA)#(mG)(fA)(mC)(fU)(mC)(fA)(mG)(fA)(mA)(fU)(mG)#(fU)#(mG)#(fC)#(mU)#(fC)#(mG)#(fG)	(fC)#(mA)#(fC)(mA)(fU)(mU)(fC)(mU)(fG)(mA)(fG)(mU)(fC)#(mU)#(fA)-TegChol
Apoe_1219	Mouse ApoE	NM_009696	P(mU)#(fA)#(mG)(fA)(mG)(fA)(mC)(fU)(mC)(fA)(mG)(fA)(mA)#(fU)#(mG)#(fU)#(mG)#(fC)#(mU)#(fC)	(fC)#(mA)#(fU)(mU)(fC)(mU)(fG)(mA)(fG)(mU)(fC)(mU)(fC)#(mU)#(fA)-TegChol
Apoe_1223	Mouse ApoE	NM_009696	P(mU)#(fU)#(mC)(fA)(mC)(fA)(mG)(fA)(mG)(fA)(mC)(fU)(mC)#(fA)#(mG)#(fA)#(mA)#(fU)#(mG)#(fU)	(fC)#(mU)#(fG)(mA)(fG)(mU)(fC)(mU)(fC)(mU)(fG)(mU)(fG)#(mA)#(fA)-TegChol
Apoe_1227	Mouse ApoE	NM_009696	P(mU)#(fU)#(mC)(fA)(mC)(fU)(mC)(fA)(mC)(fA)(mG)(fA)(mG)#(fA)#(mC)#(fU)#(mC)#(fA)#(mG)#(fA)	(fG)#(mU)#(fC)(mU)(fC)(mU)(fG)(mU)(fG)(mA)(fG)(mU)(fG)#(mA)#(fA)-TegChol
Apoe_1230	Mouse ApoE	NM_009696	P(mU)#(fG)#(mA)(fA)(mU)(fC)(mA)(fC)(mU)(fC)(mA)(fC)(mA)#(fG)#(mA)#(fG)#(mA)#(fC)#(mU)#(fC)	(fU)#(mC)#(fU)(mG)(fU)(mG)(fA)(mG)(fU)(mG)(fA)(mU)(fU)#(mC)#(fA)-TegChol
Apoe_1233	Mouse ApoE	NM_009696	P(mU)#(fU)#(mU)(fG)(mG)(fA)(mA)(fU)(mC)(fA)(mC)(fU)(mC)#(fA)#(mC)#(fA)#(mG)#(fA)#(mG)#(fA)	(fG)#(mU)#(fG)(mA)(fG)(mU)(fG)(mA)(fU)(mU)(fC)(mC)(fA)#(mA)#(fA)-TegChol

Detailed sequence and chemical modification patterns, of siRNAs. Chemical modifications are designated as follows, “#” –phosphorothioate bond, “m” – 2’-O-Methyl, “f” – 2’-Fluoro, “P” – 5’ Phosphate.

Table 2: Partially modified sense strand sequences and chemical pattern

Name	Gene	Accession Number	Sense strand
Apoe_156	Mouse ApoE	NM_009696	(fA)#(mU)#(fC)(rA)(rA)(rU)(fG)(mA)(fG)(mU)(fA)(mU)(fC)#(mC)#(fA)-TegChol
Apoe_416	Mouse ApoE	NM_009696	(fA)#(mA)#(fU)(rG)(rA)(rG)(fU)(mA)(fU)(mC)(fC)(mU)(fU)#(mC)#(fA)-TegChol
Apoe_636	Mouse ApoE	NM_009696	(fG)#(mA)#(fG)(rU)(rA)(rU)(fC)(mC)(fU)(mU)(fC)(mU)(fC)#(mC)#(fA)-TegChol
Apoe_1004	Mouse ApoE	NM_009696	(fU)#(mA)#(fU)(rC)(rC)(rU)(fU)(mC)(fU)(mC)(fC)(mU)(fG)#(mU)#(fA)-TegChol
Apoe_1032	Mouse ApoE	NM_009696	(fC)#(mC)#(fU)(rU)(rC)(rU)(fC)(mC)(fU)(mG)(fU)(mC)(fC)#(mU)#(fA)-TegChol
Apoe_1103	Mouse ApoE	NM_009696	(fC)#(mC)#(fU)(rG)(rU)(rC)(fC)(mU)(fG)(mC)(fA)(mA)(fC)#(mA)#(fA)-TegChol
Apoe_1106	Mouse ApoE	NM_009696	(fG)#(mU)#(fC)(rC)(rU)(rG)(fC)(mA)(fA)(mC)(fA)(mA)(fC)#(mA)#(fA)-TegChol
Apoe_1109	Mouse ApoE	NM_009696	(fG)#(mC)#(fA)(rA)(rC)(rA)(fA)(mC)(fA)(mU)(fC)(mC)(fA)#(mU)#(fA)-TegChol
Apoe_1112	Mouse ApoE	NM_009696	(fA)#(mA)#(fC)(rA)(rU)(rC)(fC)(mA)(fU)(mA)(fU)(mC)(fC)#(mA)#(fA)-TegChol
Apoe_1115	Mouse ApoE	NM_009696	(fA)#(mU)#(fA)(rU)(rC)(rC)(fA)(mG)(fC)(mC)(fA)(mG)(fG)#(mU)#(fA)-TegChol
Apoe_1119	Mouse ApoE	NM_009696	(fC)#(mA)#(fG)(rC)(rC)(rA)(fG)(mG)(fU)(mG)(fG)(mC)(fC)#(mC)#(fA)-TegChol
Apoe_1121	Mouse ApoE	NM_009696	(fC)#(mU)#(fC)(rA)(rA)(rG)(fC)(mA)(fC)(mC)(fU)(mC)(fU)#(mC)#(fA)-TegChol
Apoe_1123	Mouse ApoE	NM_009696	(fA)#(mG)#(fC)(rA)(rC)(rC)(fU)(mC)(fU)(mC)(fU)(mG)(fG)#(mC)#(fA)-TegChol
Apoe_1124	Mouse ApoE	NM_009696	(fU)#(mG)#(fG)(rC)(rC)(rC)(fU)(mU)(fG)(mC)(fU)(mU)(fA)#(mA)#(fA)-TegChol
Apoe_1126	Mouse ApoE	NM_009696	(fC)#(mC)#(fU)(rU)(rG)(rC)(fU)(mU)(fA)(mA)(fU)(mA)(fA)#(mA)#(fA)-TegChol
Apoe_1129	Mouse ApoE	NM_009696	(fG)#(mC)#(fU)(rU)(rA)(rA)(fU)(mA)(fA)(mA)(fG)(mA)(fU)#(mU)#(fA)-TegChol
Apoe_1134	Mouse ApoE	NM_009696	(fA)#(mA)#(fA)(rG)(rA)(rU)(fU)(mC)(fU)(mC)(fC)(mG)(fA)#(mG)#(fA)-TegChol
Apoe_1141	Mouse ApoE	NM_009696	(fU)#(mC)#(fU)(rC)(rC)(rG)(fA)(mG)(fC)(mA)(fC)(mA)(fU)#(mU)#(fA)-TegChol
Apoe_1146	Mouse ApoE	NM_009696	(fC)#(mG)#(fA)(rG)(rC)(rA)(fC)(mA)(fU)(mU)(fC)(mU)(fG)#(mA)#(fA)-TegChol
Apoe_1163	Mouse ApoE	NM_009696	(fC)#(mA)#(fU)(rU)(rC)(rU)(fG)(mA)(fG)(mU)(fC)(mU)(fC)#(mU)#(fA)-TegChol
Apoe_1167	Mouse ApoE	NM_009696	(fC)#(mU)#(fG)(rA)(rG)(rU)(fC)(mU)(fC)(mU)(fG)(mU)(fG)#(mA)#(fA)-TegChol
Apoe_1188	Mouse ApoE	NM_009696	(fG)#(mU)#(fC)(rU)(rC)(rU)(fG)(mU)(fG)(mA)(fG)(mU)(fG)#(mA)#(fA)-TegChol
Apoe_1191	Mouse ApoE	NM_009696	(fU)#(mC)#(fU)(rG)(rU)(rG)(fA)(mG)(fU)(mG)(fA)(mU)(fU)#(mC)#(fA)-TegChol
Apoe_1192	Mouse ApoE	NM_009696	(fG)#(mU)#(fG)(rA)(rG)(rU)(fG)(mA)(fU)(mU)(fC)(mC)(fA)#(mA)#(fA)-TegChol
Apoe_1196	Mouse ApoE	NM_009696	(fA)#(mU)#(fC)(rA)(rA)(rU)(fG)(mA)(fG)(mU)(fA)(mU)(fC)#(mC)#(fA)-TegChol
Apoe_1203	Mouse ApoE	NM_009696	(fA)#(mA)#(fU)(rG)(rA)(rG)(fU)(mA)(fU)(mC)(fC)(mU)(fU)#(mC)#(fA)-TegChol
Apoe_1209	Mouse ApoE	NM_009696	(fG)#(mA)#(fG)(rU)(rA)(rU)(fC)(mC)(fU)(mU)(fC)(mU)(fC)#(mC)#(fA)-TegChol
Apoe_1213	Mouse ApoE	NM_009696	(fU)#(mA)#(fU)(rC)(rC)(rU)(fU)(mC)(fU)(mC)(fC)(mU)(fG)#(mU)#(fA)-TegChol
Apoe_1213	Mouse ApoE	NM_009696	(fC)#(mC)#(fU)(rU)(rC)(rU)(fC)(mC)(fU)(mG)(fU)(mC)(fC)#(mU)#(fA)-TegChol
Apoe_1217	Mouse ApoE	NM_009696	(fC)#(mC)#(fU)(rG)(rU)(rC)(fC)(mU)(fG)(mC)(fA)(mA)(fC)#(mA)#(fA)-TegChol
Apoe_1219	Mouse ApoE	NM_009696	(fG)#(mU)#(fC)(rC)(rU)(rG)(fC)(mA)(fA)(mC)(fA)(mA)(fC)#(mA)#(fA)-TegChol
Apoe_1223	Mouse ApoE	NM_009696	(fG)#(mC)#(fA)(rA)(rC)(rA)(fA)(mC)(fA)(mU)(fC)(mC)(fA)#(mU)#(fA)-TegChol
Apoe_1227	Mouse ApoE	NM_009696	(fA)#(mA)#(fC)(rA)(rU)(rC)(fC)(mA)(fU)(mA)(fU)(mC)(fC)#(mA)#(fA)-TegChol
Apoe_1230	Mouse ApoE	NM_009696	(fA)#(mU)#(fA)(rU)(rC)(rC)(fA)(mG)(fC)(mC)(fA)(mG)(fG)#(mU)#(fA)-TegChol
Apoe_1233	Mouse ApoE	NM_009696	(fC)#(mA)#(fG)(rC)(rC)(rA)(fG)(mG)(fU)(mG)(fG)(mC)(fC)#(mC)#(fA)-TegChol

Detailed sequence and chemical modification patterns, of siRNAs. Chemical modifications are designated as follows, “#” –phosphorothioate bond, “m” – 2’-O-Methyl, “f” – 2’-Fluoro, “P” – 5’ Phosphate.

Table 3: Key Resources

Reagent	Source	Identifier
**QuantiGene Probeset**		
Mouse ApoE	Thermofisher	SB-13611
Mouse PPIB	Thermofisher	SB-10002
**Antibodies**		
Rabbit polyclonal anti-ApoE	Abcam	183597
Rabbit polyclonal anti-beta-actin	Cell Signaling Technologies	4970
Anti-NeuN	Millipore	MAB37
Anti-GFAP	Abcam	5441
**RNAscope Probe sets**		
ApoE	ACDBio	313271
HPRT	ACDBio	312951
**Commerical Assays**		
QuantiGene 2.0	Thermofisher	QS0011
RNAscope Fluorescent Multiplex Kit	ACDBio	320850
**Deposited Data**		
N2A cells	GSE45119	
Astrocytes	GSE52564	
**Primers**	Sequence	
ApoE Forward	GCTCAGACCCTGGAGGCTAA	
ApoE Reverse	CTGTTCCTCCAGCTCCTTTTTGTA	
