# Supplementary material for: Cell Type Impacts Accessibility of mRNA to Silencing by RNA Interference
Source: Mol Ther Nucleic Acids. 2020 Jun 12;21:384–93. doi: 10.1016/j.omtn.2020.06.006 (PMC7340969; doi:10.1016/j.omtn.2020.06.006)
Supplement: Document S2. Article plus Supplemental Information [file mmc2.pdf]

Molecular Therapy
Nucleic Acids
Original Article

Cell Type Impacts Accessibility of mRNA to Silencing by RNA Interference

Chantal M. Ferguson,[1] Dimas Echeverria,[1] Matthew Hassler,[1] Socheata Ly,[1] and Anastasia Khvorova[1]

[1]RNA Therapeutics Institute, University of Massachusetts Medical School, Worcester, MA 01605, USA

RNA interference (RNAi) is a potent mechanism that silences mRNA and protein expression in all cells and tissue types. RNAi is known to exert many of its functional effects in the cytoplasm, and thus, the cellular localization of target mRNA may impact observed potency. Here, we demonstrate that cell identity has a profound impact on accessibility of apolipoprotein E (*ApoE*) mRNA to RNAi. We show that, whereas both neuronal and glial cell lines express detectable *ApoE* mRNA, in neuronal cells, *ApoE* mRNA is not targetable by RNAi. Screening of a panel of thirty-five chemically modified small interfering RNAs (siRNAs) did not produce a single hit in a neuronal cell line, whereas up to fifteen compounds showed strong efficacy in glial cells. Further investigation of the cellular localization of *ApoE* mRNA demonstrates that *ApoE* mRNA is partially spliced and preferentially localized to the nucleus (∼80%) in neuronal cells, whereas more than 90% of *ApoE* mRNA is cytoplasmic in glial cells. Such an inconsistency in intracellular localization and splicing might provide an explanation for functional differences in RNAi compounds. Thus, cellular origin might have an impact on accessibility of mRNA to RNAi and should be taken into account during the screening process.

INTRODUCTION

mRNA silencing via RNA interference (RNAi) is a potent mechanism that silences gene expression in all cell and tissue types. Chemically modified small interfering RNAs (siRNAs) are small, double-stranded oligonucleotides that load into the RNA-induced silencing complex (RISC) and target mRNA for cleavage and degradation prior to translation into protein.[1] Antisense oligonucleotides (ASOs) cause mRNA silencing via both nuclear and cytoplasmic RNase H.[1] Whereas RNAi machinery is present in both the cytoplasm and the nucleus, the degree of efficacy has been shown to be much higher in the cytoplasm.[2,3] Whereas it is possible that the cellular localization of mRNA (nuclear or cytoplasmic) may impact the accessibility of mRNA to RNAi, other studies show clear examples of potent RNAi in the nucleus,[4] thus suggesting alternative mechanisms of apolipoprotein E (*ApoE*) mRNA resistance to RNAi, such as intron retention.

The mechanism of RNAi is very well characterized and understood,[5,6] with many algorithms developed for predicting siRNA efficacy.[7] In general, it is believed that the dominant factor defining siRNA activity is efficient RISC loading, followed by proper accommodation for enzymatic cleavage and product release and some contribution from target-site accessibility. Thus, compounds identified in one cellular background have a tendency to be active across cell types and tissues, with rare exceptions. As a result, the generic screening strategy relies on identification of an easily expandable cell line with reasonable target expression levels for the primary screen, following with hit validation in relevant cells and *in vivo*.

We screened a panel of fully modified siRNAs (35) targeting *ApoE* in two different cell lines—mouse neuroblastoma 2a (N2A) cells and mouse primary astrocytes—and observed stark differences in efficacy. ApoE, a member of the larger family of lipoproteins, is expressed and functions in distinct physiological compartments.[8] Systemic *ApoE* is secreted mainly by hepatocytes and facilitates lipid uptake into peripheral tissues via low-density lipoprotein (LDL) receptors.[9,10] In the central nervous system (CNS), *ApoE* is expressed by astrocytes and to a lesser extent, neurons, to transport lipids between cells and modulate the inflammatory response.[11–14] Previous studies suggest that whereas the basal expression of *ApoE* is relatively low in neurons compared to glial cells, neuronal *ApoE* is activated in response to injury or inflammation. Upon activation, incompletely spliced *ApoE* pre-mRNA may mature into mRNA and is transported from the nucleus to the cytoplasm.[11,12] This phenomenon is not unique to *ApoE*: intron retention as a mechanism for controlling gene expression is well documented and observed in neuronal cells.[15]

With the use of advanced fluorescence *in situ* hybridization (FISH), we show that whereas both cells of neuronal and glial origin express *ApoE* mRNA, the expression levels and cellular localization (nuclear versus cytoplasm) of *ApoE* vary between cell types, potentially impacting accessibility to RNAi. Furthermore, with the use of RNA sequencing (RNA-seq), we show that *ApoE* mRNA in N2A cells may not be completely spliced, suggesting that intron retention may be an additional mechanism by which *ApoE* mRNA resists silencing.

Received 31 March 2020; accepted 8 June 2020;
https://doi.org/10.1016/j.omtn.2020.06.006.

Correspondence: Anastasia Khvorova, University of Massachusetts Medical School, 368 Plantation Street, Worcester, MA 01605, USA.
E-mail: anastasia.khvorova@umassmed.edu

 © 2020 The Authors.
This is an open access article under the CC BY license (http://creativecommons.org/licenses/by/4.0/).

RESULTS

Cell Type Impacts Efficacy of siRNAs Targeting *ApoE*

We designed and synthesized a panel of thirty-five fully modified, cholesterol-conjugated siRNAs targeting all regions (5′ UTR, open reading frame [ORF], 3′ UTR) of mouse *ApoE* mRNA. Inclusion of a cholesterol conjugate allows for passive uptake of siRNAs into cells and mitigates the need for lipid-mediated transfection.[16] We tested all *ApoE*-targeting siRNAs in mouse N2A cells at a 1.5-μM concentration and observed that none of the thirty-five compounds induced silencing of *ApoE* mRNA (Figure 1A). To identify efficacious siRNAs, we designed several sequences per gene, following the rules laid out in Birmingham et al.[17] The typical hit rate for this chemical configuration in the context of advanced bioinformatics algorithms differs between different genes but ranges between 10% and 40%. Therefore, it is highly unusual that none out of thirty-five sequences would be efficacious.

For initial screening, we used a well-established and validated assay: QuantiGene. This assay serves as a high-throughput, ELISA-like method for quantifying mRNA and identifying efficacious siRNAs by measuring target RNA directly from cell or tissue lysates in a 96-well plate format.[18,19] With the use of probe sets specific for mouse *ApoE*, we observed specific detection of *ApoE* mRNA in N2A cells, with proportional increase in signal as the volume of lysate was increased (Figure 1B), defining the broad linear range of the signal.

To ensure that lack of siRNA efficacy was not due to a nonspecific signal, we confirmed *ApoE* expression in N2A cells using qRT-PCR on purified RNA. Similar to results observed using QuantiGene, *ApoE* mRNA was detectable using qRT-PCR in a concentration-dependent manner (Figure 1C). In both qRT-PCR and QuantiGene, the *ApoE* mRNA expression level was normalized to the housekeeping gene *Ppib* to control for variances in cell number or lysate volume. Additionally, we confirmed expression of processed *ApoE* in N2A cells using previously published RNA-seq datasets (Figure 1D).[20] *ApoE* mRNA was easily detected at 8.1 transcripts per million (TPM) (Figure 1D), showing typical exon/intron distribution density with exception of intron 1. Thus, *ApoE* mRNA is expressed in N2A cells, and the absence of observable efficacy with any of the thirty-five tested compounds may not be explained by lack of probe specificity.

In vivo, astrocytes have a higher expression level of *ApoE* compared to neurons. To determine if cell identity had an impact on siRNA efficacy, we screened the same thirty-five fully modified siRNAs targeting *ApoE* in mouse primary astrocytes (Figure 1E). Both QuantiGene and qRT-PCR efficiently detected *ApoE* mRNA expression with a wide linear range, albeit at a much higher level than in N2A cells (Figures 1F and 1G). RNA-seq data confirmed expression in astrocytes at substantially higher levels (17,000 TPM), with no reads mapping to the first intron (Figure 1H).[21]

In contrast to N2A cells, in mouse primary astrocytes, six (17%) and fifteen (43%) out of thirty-five siRNAs silenced *ApoE* mRNA expression by more than 90% and 50%, respectively (Figure 1E). The observed hit rate of more than 17% correlates with previous experience in screening siRNAs.

To see if a reduction in chemical modifications and increased passenger strand dissociation impacted accessibility to RNAi,[22] we synthesized the same panel of siRNAs with conserved 2′ hydroxyl ribose at positions four, five, and six of the sense strand (Table S2). Reduction in chemical modification of the sense strand did not impact the efficacy of siRNAs targeting *ApoE* in either N2A cells (Figure S2A) or mouse primary astrocytes (Figure S2B). These results suggest that resistance to degradation via RNAi may not be due to the complete chemical modification of siRNAs. Future studies are necessary to determine if completely unmodified siRNAs demonstrate similar results.

We demonstrate that whereas both N2A cells and astrocytes express *ApoE*, although at different amounts, the ability of siRNAs to access and silence *ApoE* mRNA is meaningfully affected by cell type.

We have previously shown that cellular origin has an impact on subcellular localization of huntingtin mRNA in both wild-type mice[23] and mutant models of Huntington's disease models. We found that in cells of neuronal origin, a larger portion of *Htt* mRNA was localized to the nucleus, corresponding to a lower degree of observable mRNA silencing.[23] Thus, as a next step, we evaluated *ApoE* intracellular localization in different cell types.

ApoE mRNA Expression Level and Localization Vary between Cell Type

To visually detect *ApoE* mRNA and investigate its cellular localization, we used an advanced version of FISH technology, RNAscope. RNAscope requires sequential binding of multiple Z probes that are complementary to the target mRNA, ensuring highly specific binding. Next, a branched DNA amplification system allows for the detection of single RNA molecules in cells with high resolution and sensitivity.[24] With the use of fluorescent imaging, it is possible to visualize individual mRNA foci and quantify relative nucleus or cytoplasm localization.

With the use of RNAscope, *ApoE* mRNA foci were detected in both nucleus and cytoplasm with ~5 (±4) and 2 (±3) copies in the nucleus and cytoplasm, respectively (Figures 2A and 2B). The cumulative per-cell number of foci correlated well with qRT-PCR and QuantiGene (Figures 1B and 1C). The nuclear-to-cytoplasmic ratio of *ApoE* mRNA was ~80% (Figure 2B). With the assumption that the nuclear fraction of mRNA is less accessible to RNAi in the experimental time frame (3 days), preferential nuclear localization may explain the lack of observable silencing.

In primary mouse astrocytes, consistent with qRT-PCR and QuantiGene data, RNAscope showed a high amount of *ApoE* mRNA expression in both the nucleus and the cytoplasm (Figure 2C), with >90% of mRNA showing cytoplasmic localization (Figure 2D).

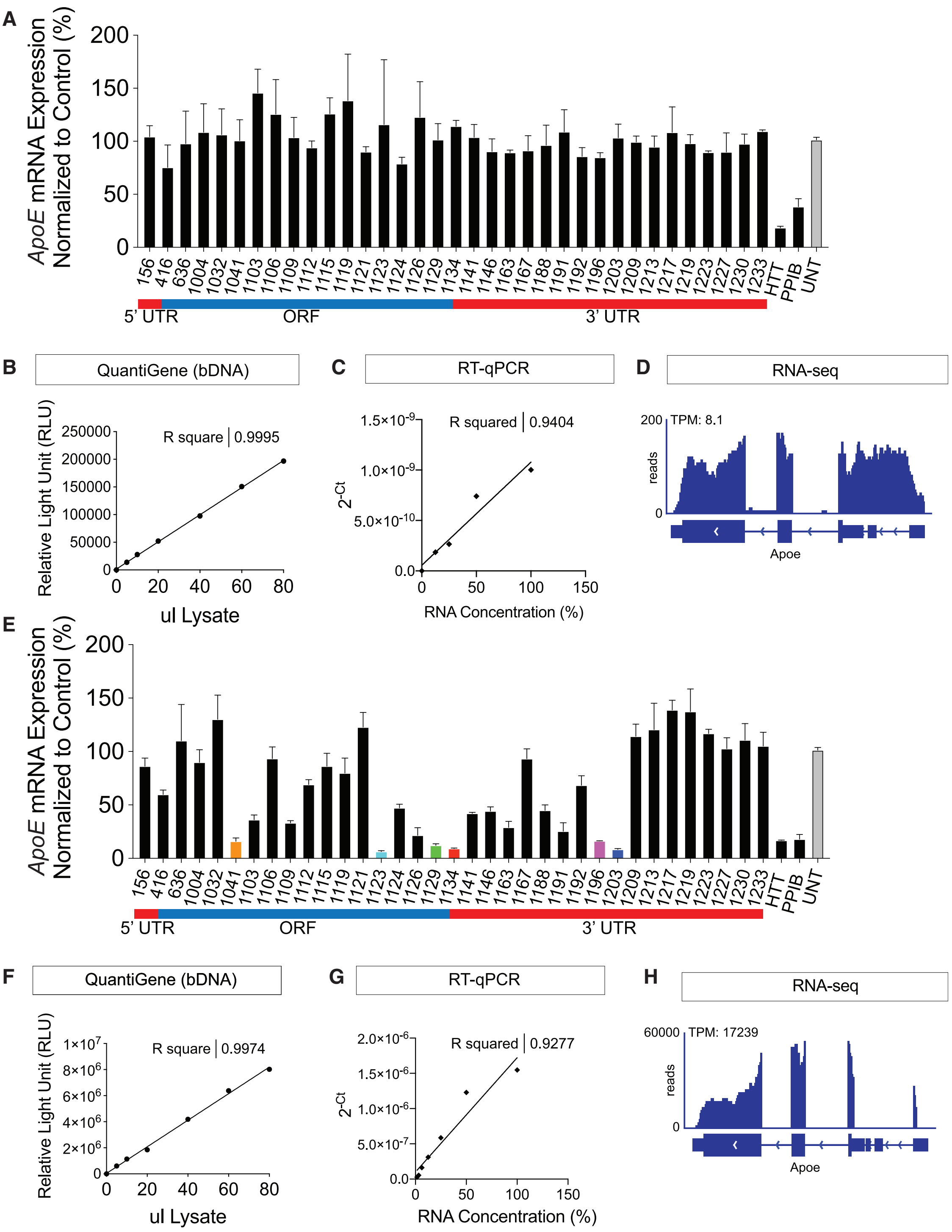

Figure 1. Efficacy of siRNAs Targeting *ApoE* Is Impacted by Cell Line Despite mRNA Expression

(A) Screen of 35 fully modified siRNAs in mouse N2A cells. Previously validated siRNAs targeting HTT and PPIB were used as positive controls for screening assays. (B and F) mRNA expression (relative light unit [RLU]) versus cell lysate volume using the QuantiGene branched DNA (bDNA) assay for mRNA expression in N2A (B) or primary astrocytes (F). (C and G) *ApoE* mRNA expression in N2A cells (C) and astrocytes (G) versus RNA concentration using qRT-PCR. (E) Screen of same 35 fully modified siRNAs in mouse primary astrocytes. (D and H) Analysis of RNA-seq datasets confirming expression of *ApoE* mRNA in N2A (D) and primary astrocytes (H).

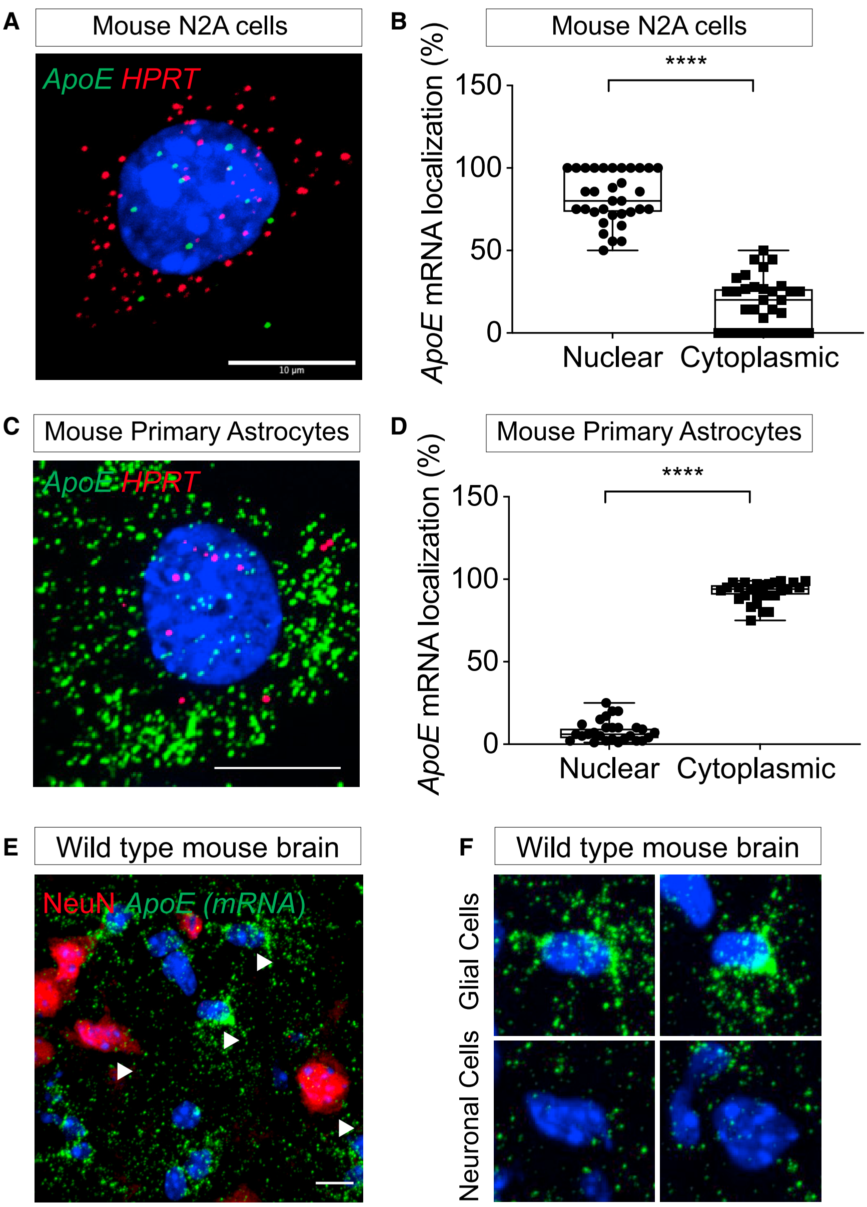

Figure 2. Increased Nuclear Localization of *ApoE* mRNA in Neuronal Cells

(A and C) *ApoE* mRNA (green) in (A) mouse N2A cells and (C) primary astrocytes (HPRT in red). (B and D) Quantification of nuclear and cytoplasmic mRNA foci in (B) N2A cells and (D) primary astrocytes (% of total foci). (E) *ApoE* mRNA (green) in wild-type mouse brain, with NeuN (red) marking neuronal cells. (F) Zoom (arrows) of wild-type mouse brain showing qualitative increase in *ApoE* mRNA in non-neuronal (NeuN-negative) cells and preferential cytoplasmic localization. Scale bars, 10 µm. Statistical analysis: t-tests using GraphPad Prism. Error bars are SD. (**** $p < 0.0001$; *** $p < 0.002$; ** $p < 0.01$; * $p < 0.05$).

To see if expression patterns were similar *in vivo*, we examined *ApoE* mRNA expression in wild-type mouse brain samples using a modified version of RNAscope/immunofluorescence. Staining for NeuN was used to define neuronal cells. Qualitatively, we observed analogous distribution of *ApoE* mRNA in neuronal and non-neuronal cells (Figures 2E and 2F), where glial cells expressed substantially higher amounts of *ApoE* mRNA with clear cytoplasmic preference.

Dose-Dependent Reduction of *ApoE* Observed in Mouse Primary Astrocytes

Visualization of mRNA silencing using RNAscope shows no detectable effect on *ApoE* expressed in N2A cells (Figures 3A and 3B), whereas an almost-complete reduction of *ApoE* in astrocytes was observed (Figures 3C and 3D). To explore the impact of cell type on RNAi efficacy in more detail, we performed dose-response studies using lead siRNA sequences: $APOE^{1134}$ and $APOE^{1203}$. Both compounds (in an eight-point dose response) showed no detectable silencing in N2A cells (Figure 3E), whereas there was clear dose-dependent reduction of *ApoE* mRNA expression in primary astrocytes (Figure 3F). In addition, we treated both N2A cells and primary astrocytes with 3 doses of lead siRNA ($APOE^{1134}$) and evaluated silencing using qRT-PCR (Figure 3H). Once again, we observed dose-dependent silencing in primary astrocytes but no silencing in N2A cells (Figure 3H), suggesting that the lack of observed silencing in N2A cells is not due to the method used to quantify mRNA. On a protein level, we observed a dose-dependent reduction of APOE protein expression in primary astrocytes (treatment with $APOE^{1134}$ versus nontargeting control [NTC]) (Figure 3G). Interestingly, despite the presence of *ApoE* mRNA (Figures 1 and 3A), the APOE protein expression in a N2A cell was below the level of detection (Figure 3G, top). Taken together, these observations are consistent with the large body of literature that supports glial cells as the primary source of CNS *ApoE* expression.[11,12,25]

Thus, N2A cells express detectable *ApoE* mRNA at ~7 foci per cell (RNAscope), and mRNA is detectable by QuantiGene, qRT-PCR, and RNA-seq (Figure 1). Even though *ApoE* mRNA is properly spliced in N2A cells, it is preferentially (~80%) localized to the nucleus (Figure 1D). Interestingly, whereas *ApoE* mRNA is expressed and detected, it is not efficiently translated, as the amount of APOE protein is below the level of detection and is not readily accessible to RNAi in all doses tested.

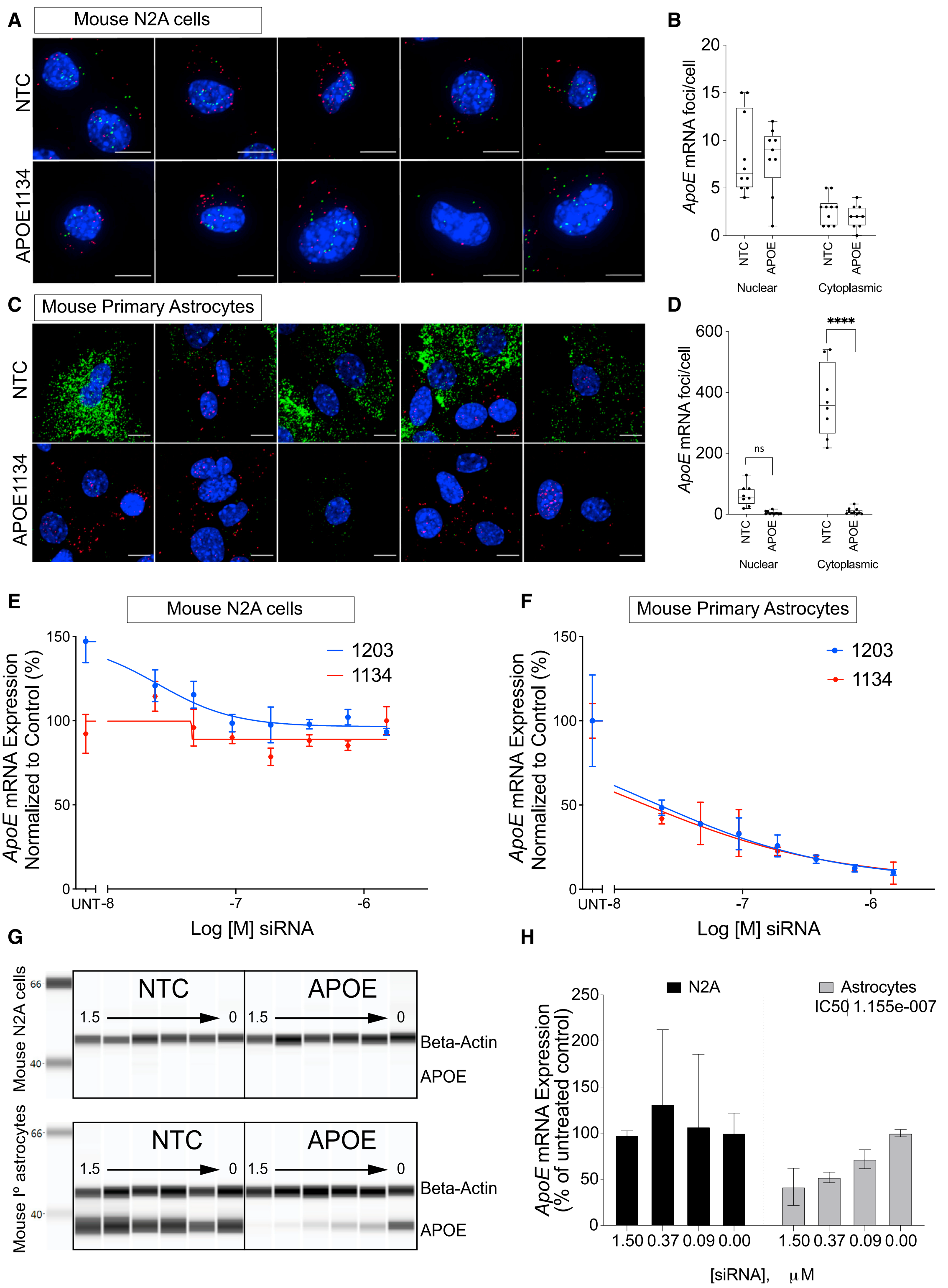

Figure 3. Dose-Dependent Reduction of *ApoE* Only in Primary Astrocytes

(A) RNAscope showing no change in *ApoE* mRNA foci between N2A cells treated with nontargeting control (NTC) and ApoE-targeting siRNA (APOE^{1134}). (B) Quantification of neuronal and cytoplasmic *ApoE* mRNA foci in N2A cells. (C) RNAscope showing reduction of *ApoE* mRNA in primary astrocytes after treatment with ApoE-targeting siRNA

(legend continued on next page)

Therefore, intron retention and subsequent intracellular localization of *ApoE* mRNA may be affected by cell type and may impact its accessibility to RNAi-mediated degradation.

DISCUSSION

In this study, we investigated the impact of different cell lines on mRNA accessibly to and efficacy of RNAi. We observed significant differences in siRNA efficacy between neuronal and astrocytic cell lines when targeting *ApoE*. We further show that neuronal cells have lower expression of *ApoE* mRNA, with predominantly nuclear localization, potentially contributing to the reduced accessibility to RNAi compared to astrocytic cells. Previous studies show that *ApoE* mRNA and protein expression levels differ between cell types and that inflammation and injury may activate neuronal *ApoE* expression.[11,12,26] Indeed, with the use of multiple assays, we found that *ApoE* mRNA expression levels are much lower in neuronal cells than astrocytic cells but still detectable using QuantiGene, qRT-PCR, and RNA-seq.

So, what is the mechanism behind *ApoE* mRNA resistance to RNAi in N2A cells? As the level of N2A *ApoE* is significantly lower compared to astrocytes, one obvious explanation is that the detection of *ApoE* mRNA is an artifact. To exclude this possibility, we evaluated *ApoE* mRNA expression using four independent methodologies: QuantiGene (signal to noise ~10), qRT-PCR (raw cycle threshold [ct] value ~29), RNA-seq (TPM: 8.1), and RNAscope (5–7 foci per cell). The efficient detection of *ApoE* mRNA by multiple experimental approaches supports the notion that the low, but detectable, expression of *ApoE* in N2A cells is not artificial.

siRNA therapeutics are becoming increasingly attractive as novel approaches for the treatment of genetically defined neurological diseases.[27] There is a body of literature on the optimal design and synthesis of fully modified siRNAs,[17,28,29] and prior to testing siRNAs in animal models, efficacious sequences can be identified and confirmed in cell-based models. However, there is minimal information available on selecting optimal cell lines for screening siRNAs.

In general, it is believed that the nature of the cell line does not bias the selection of efficacious siRNA compounds, and data generated in one cell line will be comparable with others. There are rare examples of varying single nucleotide polymorphisms between cell lines impacting RNAi,[30,31] but these sequence variations only impact the efficacy of one particular siRNA candidate, not an entire panel.

Here, we show that even though *ApoE* mRNA is readily detectable, thirty-five siRNAs targeting *ApoE* failed to induce silencing in N2A cells. However, a large fraction of these compounds were active in primary astrocytes. Furthermore, despite lack of efficacy in N2A cells, lead siRNA compounds targeting *ApoE* potently silenced *ApoE* mRNA expression below the level of detection in mouse brains.[32] Our results provide a potential explanation for discordance between *in vitro* and *in vivo* efficacy previously reported,[33] particularly if the *in vitro* characteristics do not reflect endogenous conditions. Therefore, it is crucial to select cell types that closely reflect the target cell and tissue type in order to determine the properties of RNA expression, molecular pathology, and functionality of siRNAs.

Thus, this study identifies an additional troubleshooting step during siRNA sequence screening: cell line selection and validation. Nonoptimal selection of the cell line has the potential to skew results and provide false negatives, causing *in vitro* efficacy data to not correlate well with *in vivo* efficacy. At this point, it is hard to conclude how widespread this phenomenon is. However, if the level of expression of the target mRNA is relatively low, and siRNAs fail to functionally reduce mRNA, then the selection of other cell lines or evaluation of intracellular mRNA localization might be worth considering.

One other intriguing observation is the preferential localization of neuronal *ApoE* mRNA to the nuclear compartment. The preferential nuclear localization of *ApoE* mRNA correlates with lack of detectable protein expression and limited accessibility to RNAi. In general, Ago2 is shown to be present and active both in the nucleus and cytoplasm,[34–36] but RNAi efficacy is believed to be more profound and potent in cytoplasm. Thus, altered intracellular localization might be, at least partially, responsible for lack of activity, although it is unclear what the underlying cause of the observed neuronal localization is.

Based on the RNA-seq data, N2A-expressed *ApoE* mRNA is predominately spliced (Figure 1D), which is similar to previously reported results describing nuclear localization of HTT mRNA.[37] In general, only one to two foci are detected per nucleus, which mapped to the transcriptional sites.[37] At this point, we do not have a detailed understanding of the mechanism behind the observed nuclear retention of *ApoE* mRNA in N2A cells. Studies by Xu et al.[11,12] indicated that intron 3 inclusion in neurons resulted in preferential nuclear retention and lack of protein translation. With the examination of RNA-seq data for N2A cells, we do not observe any detectable intron 3 retention (Figure 1D), and thus, it is hard to conclude if the phenomena reported here are similar to the one described by Xu et al.[12] However, we did observe intron 1 retention in RNA-seq data for N2A cells compared to primary astrocytes, suggesting an additional mechanism that may be responsible for lack of siRNA activity. Previous studies have reported that altered splicing causes nuclear retention, storage, or protection of mRNA, a mechanism that might apply here, and may impact the functionality of RNAi.[15] This phenomenon has been observed in

(APOE1134). (D) Quantification of *ApoE* mRNA foci after treatment with siRNAs. (E and F) Dose-dependent mRNA silencing of two lead siRNAs in (E) mouse N2A cells and (F) primary astrocytes. (G) Dose-dependent protein silencing in primary astrocytes (bottom) and no effect in N2A cells (top). (H) Quantification of *ApoE* mRNA after treatment with APOE[1134]. Left: N2A cells; right: primary astrocytes. Time point: 72 h. Scale bars, 10 μm. Statistical analysis: t-tests using GraphPad Prism. Error bars are SD. (**** $p < 0.0001$; *** $p < 0.002$; ** $p < 0.01$; * $p < 0.05$).

neuronal cells,[15] with certain transcripts evading endogenous decay methods and intron splicing occurring in response to stress signals[15] and potentially contributing to neurodegenerative disease phenotypes.[38]

Further studies are necessary to investigate the implication that the majority of the detectable mRNA in neuronal-like cells may be in an inactive state,[12] that is, both not being translated into protein, and is inaccessible to RNAi, potentially due to intron retention. In addition, further investigation of the specific localization of *ApoE* mRNA, i.e., localization with ribonucleoproteins (RNPs), RNA binding proteins (RBPs), or processing (P)-bodies,[39–41] may provide insight into the mechanisms behind resistance to RNAi observed here.

The utility of an inactive pool of neuronal *ApoE* mRNA remains unclear and warrants further investigation; however, we show for the first time the impact of this phenomenon on the development and efficacy of novel therapeutic strategies, such as RNAi.

It was recently shown that ApoE4 worsens tau pathology in human-derived cell lines and that the toxicity is conferred specifically by neuronal *ApoE*.[26] Thus, the development of *ApoE*-targeted therapeutics may require modalities that modulate *ApoE* in all cell types (astroglia, microglia, neurons, etc.) in the brain in order to see improvement in neuropathology and cognition. Indeed, only complete genetic knockout of brain *ApoE* (compared to 50% reduction with ASOs) in mouse models of Alzheimer's disease (AD) resulted in pathology improvement.[42–45] Thus, the understanding of how cell identity and cell state may impact the response to these therapeutics is increasingly important and may have implications that reach further than cell-based screening studies and stretch throughout the drug-development process.

MATERIALS AND METHODS

Ethics Statement

All national regulations and guidelines for the human care and use of animals (including the timed pregnant mice used to obtain primary neurons) were followed, and the animal procedures were approved by the University of Massachusetts Medical School Institutional Animal Care and Use Committee (IACUC; Protocol #A2411).

Design and Synthesis of Chemically Modified siRNAs

We designed a panel of thirty-five siRNA compounds targeting the mouse *ApoE* gene. The siRNA sequences span the entirety of the *ApoE* mRNA and were designed according to the guidelines laid out in Birmingham et al.[17] Factors influencing the selection of sequences include optimal GC content, target specificity, low seed complement frequency, removal of sequences containing toxic motifs, and removal of sequence containing microRNA (miRNA) seeds.

Oligonucleotide Synthesis

Oligonucleotides were synthesized using modified (2′-fluoro [2′-F], 2′-O-methyl [2′-O-Me]) phosphoramidites with standard protecting groups (ChemGenes). Phosphoramidite solid-phase synthesis was done on a MerMade 12 (BioAutomation) and Dr. Oligo 48 (Biolytic) using modified protocols. Unconjugated oligonucleotides were synthesized on controlled pore glass (CPG) functionalized with a long-chain alkyl amine (LCAA) and unylinker terminus (ChemGenes). Cholesterol-conjugated oligonucleotides were grown with the cholesterol moiety bound to a tetraethylenglycol (TEG) attached through a succinate linker to LCAA-CPG support (ChemGenes). Phosphoramidites were prepared at 0.1 M in anhydrous acetonitrile (ACN), with added dry 15% dimethylformamide (DMF) in the 2′-O-ME U amidite. 5-(benzylthio)-1H-tetrazole (BTT) was used as the activator at 0.25 M. Detritylations were performed using 3% trichloroacetic acid in dichloromethane (DCM). Capping was done with nontetrahydrofuran-containing reagents CAP A, 20% n-methylimidazole in ACN; and CAP B, 20% acetic anhydride (Ac2O), 30% 2,6-lutidine in ACN (synthesis reagents were purchased at American International Chemical [AIC]). Sulfurization was performed with 0.1 M solution of 3-[(dimethylaminomethylene)amino]-3H-1,2,4-dithiazole-5-thione (DDTT) in pyridine (ChemGenes) for 3 min. Phosphoramidite coupling times were 3 min for all amidites used.

Deprotection and Purification of Oligonucleotides

All oligonucleotides were cleaved and deprotected using ammonium hydroxide and 40% aqueous (aq.) methylamine (AMA) in a 1:1 ratio for 2 h at room temperature. The oligonucleotide solutions were then filtered to remove the CPG from the cleaved oligo. The filtrate was then cooled with dry ice and then dried under vacuum in a SpeedVac. The resulting pellets were resuspended in 5% ACN in water. The purification of the unconjugated strands were performed on an Agilent 1200 system, equipped with a Source 15Q anion exchange resin (GE Healthcare; 10 × 100 mm custom-packed column), using the following conditions: eluent A, 20% ACN, 20 mM sodium acetate (pH 5); eluent B, 1 M sodium perchlorate in 20% ACN; gradient, 0% B 2 min, 35% B 12 min, clean, and re-equilibration to initial conditions 6 min. Purification of cholesterol-conjugated strands was performed on the same equipment but equipped with a PRP-C18 (Hamilton), a polymer reverse-phase column (10 × 100 mm), using the following conditions: eluent A, 50 mM sodium acetate in 5% ACN; eluent B, ACN; gradient, 0% B 2 min, 0%–40% B 1 min, 40%–70% B 9 min, clean, and re-equilibration 6 min. Temperature 70 °C and flow rate 40 mL/min were the same in both cases. Peaks were monitored at 260 nm. The pure oligonucleotide fractions were collected, individually characterized by liquid chromatography-mass spectrometry (LC-MS), combined, frozen, and dried in a SpeedVac overnight. Oligonucleotides were resuspended in 5% ACN and desalted through fine Sephadex G-25 (GE Healthcare; 10 × 200 mm custom-packed column) and lyophilized. All reagents mentioned above were purchased from Sigma-Aldrich and used as per the manufacturer's instructions, unless otherwise stated.

LC-MS Analysis of Oligonucleotides

The identity of oligonucleotides was established by LC-MS analysis on an Agilent 6530 accurate mass Quadrupole Time of Flight (Q-TOF) LC-MS, using the following conditions: buffer A, 100 mM

hexafluoroisopropanol (HFIP)/9 mM triethylamine (TEA) in LC-MS-grade water; buffer B, 100 mM HFIP/9 mM TEA in LC-MS-grade methanol; column, Agilent AdvanceBio Oligonucleotides C18; gradient unconjugated strands 0% B 1 min, 0%–40% B 8 min, clean, and re-equilibration 4 min; cholesterol-conjugated strands, 0% B 1 min, 0%–50% B 0.5 min, 50%–100% B 8 min, clean, and re-equilibration 4 min; temperature 45 °C and flow rate 0.5 mL/min were the same in both cases. LC peaks were monitored at 260 nm. MS parameters were the following: source, electrospray ionization; ion polarity, negative mode; range, 100–3,200 mass-to-charge ratio (*m/z*); scan rate, 2 spectra s^{-1}; capillary voltage, 4,000; fragmentor, 180 V. All reagents mentioned above were purchased from Sigma-Aldrich and used as per the manufacturer's instructions, unless otherwise stated.

Cell Culture

N2A cells (ATCC; CLL-131) were maintained in Eagle's minimum essential medium (ATCC; #30-2003), supplemented with 10% fetal bovine serum (FBS; Gibco, Carlsbad, CA, USA; #26140) and 100 U/mL penicillin/streptomycin (Invitrogen, Carlsbad, CA, USA; #15140), and grown at 37°C and 5% CO_2. Cells were split every 2–5 days.

Preparation of Primary Astrocytes

Primary cortical astrocytes were obtained from C57BL/6J mouse embryos at embryonic day 15. Pregnant C57BL/6J females were anesthetized by inhalation of isoflurane, followed by cervical dislocation. Embryos were removed and transferred to a Petri dish with ice-cold Dulbecco's modified Eagle's medium. Brains were removed and meninges detached. After isolation of cortices, brain tissue was mechanically disrupted using a surgical scalpel. Cortices were then placed in 1.5 mL of TrypLE (Thermo Fisher Scientific; #12604013) and incubated for 25 min at 37°C and 5% CO_2. The cortices were dissociated with repetitive pipetting, as described above, and plated in T-75 cm^2 flasks in DMEM, supplemented with 10% FBS and 10 ng/μL of mouse epidermal growth factor. Astrocytes were allowed to differentiate over 2 passages, cell identity was confirmed using glial markers (glial fibrillary acidic protein [GFAP]), and cells were used in assays. Dividing primary astrocytes were split every 5–7 days and discarded after 10 passages.

Delivery and Silencing with siRNAs

Cells were plated in respective media, supplemented with 6% FBS at 10,000 cells per well (50 μL/well), in 96-well tissue-culture plates. siRNA was diluted to twice the final concentration in Opti-MEM (Gibco; #31985-088). 50 μL siRNA was added to 50 μL of cells, resulting in 3% FBS in the media. Cells were incubated for 72 h at 37°C and 5% CO_2. Primary screens were performed at 1.5 μM compound dose.

mRNA Quantification: QuantiGene

mRNA and mRNA silencing were quantified using the QuantiGene 2.0 Assay (Affymetrix; #QS0011) and as described in Coles et al.[19] and Alterman et al.[18] Cells were prepared, as previously described, and probe sets were diluted, as specified in the Affymetrix protocol. The following probe sets were used: mouse ApoE (SB-13611) and mouse PPIB (SB-10002). Briefly, RNA-specific probe sets were added to precoated plates, followed by sample lysates. The probe sets and samples hybridized overnight, followed by three amplification steps that provided a high signal-to-noise ratio.

mRNA Quantification: qRT-PCR

RNA was purified from cells or tissue using QIAGEN RNeasy (-QIAGEN; #74194) and performed as specified by the manufacturer. cDNA was generated from up to 1 μg of purified RNA using the High-Capacity Reverse Transcription Kit (Thermo Fisher Scientific; #4368813). qPCR was performed using iTaq Universal SYBR Green Supermix (Bio-Rad; #1725120) following the manufacturer's instructions. Primers for mouse *ApoE* can be found in Table S3.

Analysis of RNA-Seq Datasets

Previously published datasets were used to analyze *ApoE* expression in N2A cells (GSE45119)[20] and primary mouse cortex (GSE52564)[21]. RNA-seq analysis was performed using the RNA-seq pipeline on DolphinNext (https://dolphinnext.umassmed.edu/).[46] Briefly, raw reads were aligned to the mm10 genome using Spliced Transcripts Alignment to a Reference (STAR),[47] TPM quantification was done using RNA-Seq by Expectation-Maximization (RSEM),[48] and quality control was analyzed using an RNA-seq Quality Control Package (RSeQC).[49] Data were visualized using Integrative Genomics Viewer (IGV).[50,51]

RNAscope FISH and FISH-Immunofluorescence (IF)

RNAscope probe sets for mouse *ApoE* and mouse *Hprt* were obtained from ACDBio (#313271; #312951). RNAscope, using the RNAscope Fluorescent Multiplex Kit (ACDBio; #320850), was performed following the manufacturer's instructions. Dual FISH-IF was used to detect NeuN and GFAP. RNAscope was performed as described in the protocol, followed directly by IF. Samples were incubated for 1 h in blocking solution (2% normal goat serum, 0.01% Triton X in PBS) at room temperature. Samples were washed 3 times for 5 min in PBS and incubated in primary antibodies diluted in blocking solution (anti-NeuN 1:200 #MAB377; anti-GFAP). Slides were washed 3 times for 5 min in PBS and incubated for 1 h at room temperature in Alexa Fluor secondary antibodies (1:800; Thermo Fisher Scientific). Samples were washed 3 times for 5 min in PBS, mounted in ProLong Diamond Antifade (Thermo Fisher Scientific; #P10144), and dried overnight.

Imaging

Images were acquired with a CSU10B Spinning Disk Confocal System scan head (Solamere Technology Group, Salt Lake City, UT, USA), mounted on a TE-200E2 inverted microscope (Nikon, Tokyo, Japan) with a 100× Plan/Apochromatic (APO) oil-immersion objective and a CoolSNAP HQ2 camera (Roper Technologies, Sarasota, FL, USA). z stacks were acquired in three different channels. Images were processed using ImageJ software.

Western Blots

Western blots were performed using Wes by ProteinSimple, as described in Alterman et al.[32] Briefly, cell lysates were prepared in

radioimmunoprecipitation assay (RIPA) buffer with protease inhibitor cocktail. Total protein was quantified using the standard Bradford assay. 1.2 μg of lysate, diluted in 0.1× sample buffer, was loaded into the 16- to 230-kDa assay system. Anti-ApoE antibody (Abcam; 183597) was diluted 1:200 in antibody dilution buffer, and anti-beta-actin antibody was diluted 1:25 in antibody dilution buffer. The assay was performed according to the manufacturer's instructions.

Statistics

All statistics were performed using GraphPad Prism 8.

SUPPLEMENTAL INFORMATION

Supplemental Information can be found online at https://doi.org/10.1016/j.omtn.2020.06.006.

AUTHOR CONTRIBUTIONS

C.M.F. and A.K. conceived of the project. C.M.F., D.E., M.H., and A.K. contributed to the experimental design. C.M.F., D.E., S.L., and M.H. contributed experimentally. D.E. and M.H. synthesized compounds. C.M.F. and A.K. wrote the manuscript.

CONFLICTS OF INTEREST

The authors declare no competing interests.

ACKNOWLEDGMENTS

This work was supported by NIH 5R01 NS10433-03, ADDF 20170101/Rogaev, and National Research Service award 1F30AG066373-01 and NIH T32GM107000.

REFERENCES

1. Watts, J.K., and Corey, D.R. (2012). Silencing disease genes in the laboratory and the clinic. J. Pathol. *226*, 365–379.
2. Gagnon, K.T., Li, L., Chu, Y., Janowski, B.A., and Corey, D.R. (2014). RNAi factors are present and active in human cell nuclei. Cell Rep. *6*, 211–221.
3. Kalantari, R., Chiang, C.M., and Corey, D.R. (2016). Regulation of mammalian transcription and splicing by Nuclear RNAi. Nucleic Acids Res. *44*, 524–537.
4. Lennox, K.A., and Behlke, M.A. (2016). Cellular localization of long non-coding RNAs affects silencing by RNAi more than by antisense oligonucleotides. Nucleic Acids Res. *44*, 863–877.
5. Mello, C.C., and Conte, D., Jr. (2004). Revealing the World of RNA Interference. Nature *431*, 338–342.
6. Shen, X., and Corey, D.R. (2018). Chemistry, mechanism and clinical status of antisense oligonucleotides and duplex RNAs. Nucleic Acids Res. *46*, 1584–1600.
7. Schramm, G., and Ramey, R. (2005). siRNA design including secondary structure target site prediction. Nat. Methods *2*, 632.
8. Lane-Donovan, C., Wong, W.M., Durakoglugil, M.S., Wasser, C.R., Jiang, S., Xian, X., and Herz, J. (2016). Genetic Restoration of Plasma ApoE Improves Cognition and Partially Restores Synaptic Defects in ApoE-Deficient Mice. J. Neurosci. *36*, 10141–10150.
9. Brown, M.S., and Goldstein, J.L. (1986). A receptor-mediated pathway for cholesterol homeostasis. Science *232*, 34–47.
10. Mahley, R.W., and Innerarity, T.L. (1983). Lipoprotein receptors and cholesterol homeostasis. Biochim. Biophys. Acta *737*, 197–222.
11. Xu, Q., Bernardo, A., Walker, D., Kanegawa, T., Mahley, R.W., and Huang, Y. (2006). Profile and regulation of apolipoprotein E (ApoE) expression in the CNS in mice with targeting of green fluorescent protein gene to the ApoE locus. J. Neurosci. *26*, 4985–4994.
12. Xu, Q., Walker, D., Bernardo, A., Brodbeck, J., Balestra, M.E., and Huang, Y. (2008). Intron-3 retention/splicing controls neuronal expression of apolipoprotein E in the CNS. J. Neurosci. *28*, 1452–1459.
13. Liu, C.C., Liu, C.C., Kanekiyo, T., Xu, H., and Bu, G. (2013). Apolipoprotein E and Alzheimer disease: risk, mechanisms and therapy. Nat. Rev. Neurol. *9*, 106–118.
14. Yamazaki, Y., Zhao, N., Caulfield, T.R., Liu, C.C., and Bu, G. (2019). Apolipoprotein E and Alzheimer disease: pathobiology and targeting strategies. Nat. Rev. Neurol. *15*, 501–518.
15. Mauger, O., Lemoine, F., and Scheiffele, P. (2016). Targeted Intron Retention and Excision for Rapid Gene Regulation in Response to Neuronal Activity. Neuron *92*, 1266–1278.
16. Ly, S., Navaroli, D.M., Didiot, M.C., Cardia, J., Pandarinathan, L., Alterman, J.F., Fogarty, K., Standley, C., Lifshitz, L.M., Bellve, K.D., et al. (2017). Visualization of self-delivering hydrophobically modified siRNA cellular internalization. Nucleic Acids Res. *45*, 15–25.
17. Birmingham, A., Anderson, E., Sullivan, K., Reynolds, A., Boese, Q., Leake, D., Karpilow, J., and Khvorova, A. (2007). A protocol for designing siRNAs with high functionality and specificity. Nat. Protoc. *2*, 2068–2078.
18. Alterman, J.F., Coles, A.H., Hall, L.M., Aronin, N., Khvorova, A., and Didiot, M.C. (2017). A High-throughput Assay for mRNA Silencing in Primary Cortical Neurons *in vitro* with Oligonucleotide Therapeutics. Bio Protoc. *7*, e2501.
19. Coles, A.H., Osborn, M.F., Alterman, J.F., Turanov, A.A., Godinho, B.M., Kennington, L., Chase, K., Aronin, N., and Khvorova, A. (2016). A High-Throughput Method for Direct Detection of Therapeutic Oligonucleotide-Induced Gene Silencing In Vivo. Nucleic Acid Ther. *26*, 86–92.
20. Han, A., Stoilov, P., Linares, A.J., Zhou, Y., Fu, X.D., and Black, D.L. (2014). De novo prediction of PTBP1 binding and splicing targets reveals unexpected features of its RNA recognition and function. PLoS Comput. Biol. *10*, e1003442.
21. Zhang, Y., Chen, K., Sloan, S.A., Bennett, M.L., Scholze, A.R., O'Keeffe, S., Phatnani, H.P., Guarnieri, P., Caneda, C., Ruderisch, N., et al. (2014). An RNA-sequencing transcriptome and splicing database of glia, neurons, and vascular cells of the cerebral cortex. J. Neurosci. *34*, 11929–11947.
22. Matranga, C., Tomari, Y., Shin, C., Bartel, D.P., and Zamore, P.D. (2005). Passenger-strand cleavage facilitates assembly of siRNA into Ago2-containing RNAi enzyme complexes. Cell *123*, 607–620.
23. Didiot, M.C., Ferguson, C.M., Ly, S., Coles, A.H., Smith, A.O., Bicknell, A.A., Hall, L.M., Sapp, E., Echeverria, D., Pai, A.A., et al. (2018). Nuclear Localization of Huntingtin mRNA Is Specific to Cells of Neuronal Origin. Cell Rep. *24*, 2553–2560.e5.
24. Wang, F., Flanagan, J., Su, N., Wang, L.C., Bui, S., Nielson, A., Wu, X., Vo, H.T., Ma, X.J., and Luo, Y. (2012). RNAscope: a novel in situ RNA analysis platform for formalin-fixed, paraffin-embedded tissues. J. Mol. Diagn. *14*, 22–29.
25. Theendakara, V., Peters-Libeu, C.A., Spilman, P., Poksay, K.S., Bredesen, D.E., and Rao, R.V. (2016). Direct Transcriptional Effects of Apolipoprotein E. J. Neurosci. *36*, 685–700.
26. Wang, C., Najm, R., Xu, Q., Jeong, D.E., Walker, D., Balestra, M.E., Yoon, S.Y., Yuan, H., Li, G., Miller, Z.A., et al. (2018). Gain of toxic apolipoprotein E4 effects in human iPSC-derived neurons is ameliorated by a small-molecule structure corrector. Nat. Med. *24*, 647–657.
27. Tabrizi, S.J., Leavitt, B.R., Landwehrmeyer, G.B., Wild, E.J., Saft, C., Barker, R.A., Blair, N.F., Craufurd, D., Priller, J., Rickards, H., et al.; Phase 1–2a IONIS-HTTRx Study Site Teams (2019). Targeting Huntingtin Expression in Patients with Huntington's Disease. N. Engl. J. Med. *380*, 2307–2316.
28. Fakhr, E., Zare, F., and Teimoori-Toolabi, L. (2016). Precise and efficient siRNA design: a key point in competent gene silencing. Cancer Gene Ther. *23*, 73–82.
29. Reynolds, A., Leake, D., Boese, Q., Scaringe, S., Marshall, W.S., and Khvorova, A. (2004). Rational siRNA design for RNA interference. Nat. Biotechnol. *22*, 326–330.
30. Jackson, A.L., Bartz, S.R., Schelter, J., Kobayashi, S.V., Burchard, J., Mao, M., Li, B., Cavet, G., and Linsley, P.S. (2003). Expression Profiling Reveals Off-Target Gene Regulation by RNAi. Nat. Biotechnol. *21*, 635–637.

31. Huppi, K., Martin, S.E., and Caplen, N.J. (2005). Defining and assaying RNAi in mammalian cells. Mol. Cell *17*, 1–10.

32. Alterman, J.F., Godinho, B.M.D.C., Hassler, M.R., Ferguson, C.M., Echeverria, D., Sapp, E., Haraszti, R.A., Coles, A.H., Conroy, F., Miller, R., et al. (2019). A divalent siRNA chemical scaffold for potent and sustained modulation of gene expression throughout the central nervous system. Nat. Biotechnol. *37*, 884–894.

33. Mariot, V., Joubert, R., Hourdé, C., Féasson, L., Hanna, M., Muntoni, F., Maisonobe, T., Servais, L., Bogni, C., Le Panse, R., et al. (2017). Downregulation of myostatin pathway in neuromuscular diseases may explain challenges of anti-myostatin therapeutic approaches. Nat. Commun. *8*, 1859.

34. Gagnon, K.T., Li, L., Janowski, B.A., and Corey, D.R. (2014). Analysis of nuclear RNA interference in human cells by subcellular fractionation and Argonaute loading. Nat. Protoc. *9*, 2045–2060.

35. Kalantari, R., Hicks, J.A., Li, L., Gagnon, K.T., Sridhara, V., Lemoff, A., Mirzaei, H., and Corey, D.R. (2016). Stable association of RNAi machinery is conserved between the cytoplasm and nucleus of human cells. RNA *22*, 1085–1098.

36. Matsui, M., Li, L., Janowski, B.A., and Corey, D.R. (2015). Reduced Expression of Argonaute 1, Argonaute 2, and TRBP Changes Levels and Intracellular Distribution of RNAi Factors. Sci. Rep. *5*, 12855.

37. Didiot, M.C., Ferguson, C.M., Ly, S., Coles, A.H., Smith, A.O., Bicknell, A.A., Hall, L.M., Sapp, E., Echeverria, S., Pai, A.A., et al. (2018). Nuclear Localization of Huntingtin mRNA Is Specific to Cells of Neuronal Origin. Cell Rep. *24*, 2553–2560.e5.

38. Polymenidou, M., Lagier-Tourenne, C., Hutt, K.R., Huelga, S.C., Moran, J., Liang, T.Y., Ling, S.C., Sun, E., Wancewicz, E., Mazur, C., et al. (2011). Long pre-mRNA depletion and RNA missplicing contribute to neuronal vulnerability from loss of TDP-43. Nat. Neurosci. *14*, 459–468.

39. Liu, J., Valencia-Sanchez, M.A., Hannon, G.J., and Parker, R. (2005). MicroRNA-dependent localization of targeted mRNAs to mammalian P-bodies. Nat. Cell Biol. *7*, 719–723.

40. Kulkarni, M., Ozgur, S., and Stoecklin, G. (2010). On track with P-bodies. Biochem. Soc. Trans. *38*, 242–251.

41. Eulalio, A., Behm-Ansmant, I., Schweizer, D., and Izaurralde, E. (2007). P-body formation is a consequence, not the cause, of RNA-mediated gene silencing. Mol. Cell. Biol. *27*, 3970–3981.

42. Huynh, T.-P.V., Liao, F., Francis, C.M., Robinson, G.O., Serrano, J.R., Jiang, H., Roh, J., Finn, M.B., Sullivan, P.M., Esparza, T.J., et al. (2017). Age-Dependent Effects of apoE Reduction Using Antisense Oligonucleotides in a Model of β-amyloidosis. Neuron *96*, 1013–1023.e4.

43. Holtzman, D.M., Bales, K.R., Tenkova, T., Fagan, A.M., Parsadanian, M., Sartorius, L.J., Mackey, B., Olney, J., McKeel, D., Wozniak, D., et al. (2000). Apolipoprotein E Isoform-Dependent Amyloid Deposition and Neuritic Degeneration in a Mouse Model of Alzheimer's Disease. Proc. Natl. Acad. Sci. USA *97*, 2892–2897.

44. Holtzman, D.M., Bales, K.R., Wu, S., Bhat, P., Parsadanian, M., Fagan, A.M., Chang, L.K., Sun, Y., and Paul, S.M. (1999). Expression of Human Apolipoprotein E Reduces Amyloid-Beta Deposition in a Mouse Model of Alzheimer's Disease. J. Clin. Invest. *103*, R15–R21.

45. Kim, J., Jiang, H., Park, S., Eltorai, A.E., Stewart, F.R., Yoon, H., Basak, J.M., Finn, M.B., and Holtzman, D.M. (2011). Haploinsufficiency of human APOE reduces amyloid deposition in a mouse model of amyloid-β amyloidosis. J. Neurosci. *31*, 18007–18012.

46. Yukselen, O., Turkyilmaz, O., Ozturk, A.R., Garber, M., and Kucukural, A. (2019). DolphinNext: A graphical user interface for creating, deploying and executing Nextflow pipelines. bioRxiv. https://doi.org/10.1101/689539.

47. Dobin, A., Davis, C.A., Schlesinger, F., Drenkow, J., Zaleski, C., Jha, S., Batut, P., Chaisson, M., and Gingeras, T.R. (2013). STAR: ultrafast universal RNA-seq aligner. Bioinformatics *29*, 15–21.

48. Li, B., and Dewey, C.N. (2011). RSEM: accurate transcript quantification from RNA-Seq data with or without a reference genome. BMC Bioinformatics *12*, 323.

49. Wang, L., Wang, S., and Li, W. (2012). RSeQC: quality control of RNA-seq experiments. Bioinformatics *28*, 2184–2185.

50. Robinson, J.T., Thorvaldsdóttir, H., Winckler, W., Guttman, M., Lander, E.S., Getz, G., and Mesirov, J.P. (2011). Integrative genomics viewer. Nat. Biotechnol. *29*, 24–26.

51. Thorvaldsdóttir, H., Robinson, J.T., and Mesirov, J.P. (2013). Integrative Genomics Viewer (IGV): high-performance genomics data visualization and exploration. Brief. Bioinform. *14*, 178–192.

OMTN, Volume *21*

Supplemental Information

Cell Type Impacts Accessibility of mRNA to Silencing by RNA Interference

Chantal M. Ferguson, Dimas Echeverria, Matthew Hassler, Socheata Ly, and Anastasia Khvorova

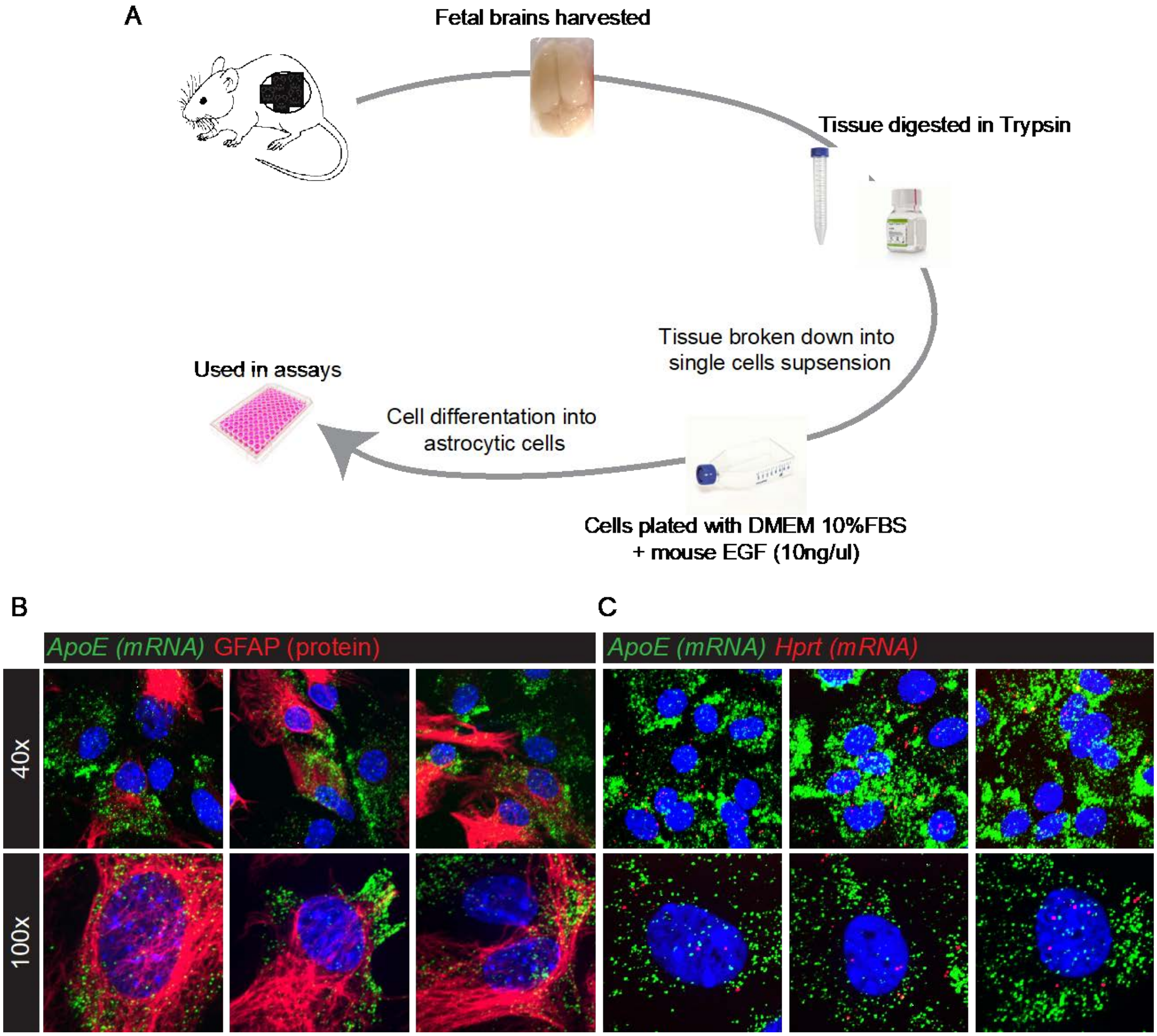

Supplementary Figure 1: Preparation of primary mouse astrocytes. (A) Workflow for harvest and preparation. (B) Primary mouse astrocytes co-stained for *ApoE* mRNA (green) (RNAscope) and GFAP protein (red) (IF) at 40x and 100x magnification confirming astrocyte lineage. (C) Primary mouse astrocytes co-stained for *ApoE* (green) and *HPRT* (red) mRNA (RNAscope) at 40x and 100x magnification.

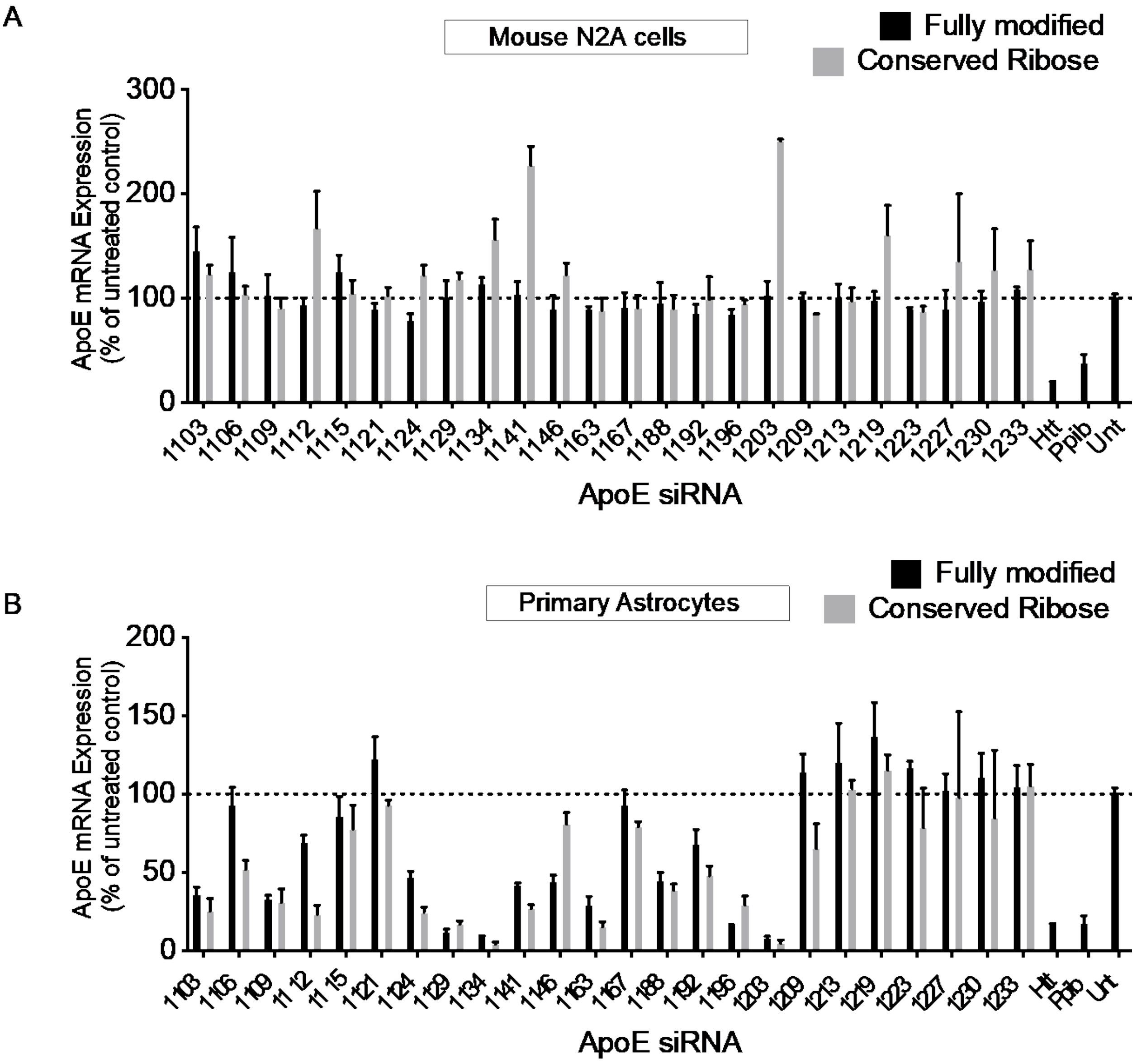

Supplementary Figure 2: Conservation of 2'OH (Ribose) does not impact RNAi efficacy. (A). Screen using fully modified (black) and partially modified (grey) siRNAs targeting *ApoE* in N2A cells. (B) Screen using fully modified (black) and partially modified (grey) siRNAs targeting *ApoE* in mouse primary astrocytes. Dose: 1.5 µM; 72 -hour timepoint; mRNA quantified using QuantiGene.

Table 1: siRNA sequences and chemical modifications

Name	Gene	Accession Number	Anti-sense strand	Sense strand
Apoe_156	Mouse ApoE	NM_009696	P(mU)#(fC)#(mA)(fA)(mU)(fU)(mG)(fU)(mG)(fA)(mU)(fU)(mG)#(fG)#(mC)#(fC)#(mA)#(fG)#(mU)#(fC)	(fG)#(mC)#(fC)(mA)(fA)(mU)(fC)(mA)(fC)(mA)(fA)(mU)(fU)#(mG)#(fA)-TegChol
Apoe_416	Mouse ApoE	NM_009696	P(mU)#(fU)#(mU)(fU)(mG)(fU)(mA)(fA)(mG)(fC)(mC)(fU)(mU)#(fU)#(mA)#(fC)#(mU)#(fU)#(mC)#(fC)	(fU)#(mA)#(fA)(mA)(fG)(mG)(fC)(mU)(fU)(mA)(fC)(mA)(fA)#(mA)#(fA)-TegChol
Apoe_636	Mouse ApoE	NM_009696	P(mU)#(fU)#(mC)(fA)(mA)(fG)(mC)(fG)(mC)(fU)(mU)(fG)(mC)#(fG)#(mC)#(fA)#(mU)#(fC)#(mU)#(fU)	(fG)#(mC)#(fG)(mC)(fA)(mA)(fG)(mC)(fG)(mC)(fU)(mU)(fG)#(mA)#(fA)-TegChol
Apoe_1004	Mouse ApoE	NM_009696	P(mU)#(fG)#(mU)(fC)(mU)(fU)(mC)(fC)(mA)(fC)(mU)(fA)(mU)#(fU)#(mG)#(fG)#(mC)#(fU)#(mC)#(fG)	(fC)#(mA)#(fA)(mU)(fA)(mG)(fU)(mG)(fG)(mA)(fA)(mG)(fA)#(mC)#(fA)-TegChol
Apoe_1032	Mouse ApoE	NM_009696	P(mU)#(fC)#(mC)(fA)(mU)(fC)(mA)(fG)(mG)(fU)(mU)(fU)(mG)#(fC)#(mC)#(fC)#(mA)#(fC)#(mU)#(fG)	(fG)#(mG)#(fC)(mA)(fA)(mA)(fC)(mC)(fU)(mG)(fA)(mU)(fG)#(mG)#(fA)-TegChol
Apoe_1103	Mouse ApoE	NM_009696	P(mU)#(fG)#(mG)(fA)(mU)(fA)(mC)(fU)(mC)(fA)(mU)(fU)(mG)#(fA)#(mU)#(fU)#(mC)#(fU)#(mC)#(fC)	(fA)#(mU)#(fC)(mA)(fA)(mU)(fG)(mA)(fG)(mU)(fA)(mU)(fC)#(mC)#(fA)-TegChol
Apoe_1106	Mouse ApoE	NM_009696	P(mU)#(fG)#(mA)(fA)(mG)(fG)(mA)(fU)(mA)(fC)(mU)(fC)(mA)#(fU)#(mU)#(fG)#(mA)#(fU)#(mU)#(fC)	(fA)#(mA)#(fU)(mG)(fA)(mG)(fU)(mA)(fU)(mC)(fC)(mU)(fU)#(mC)#(fA)-TegChol
Apoe_1109	Mouse ApoE	NM_009696	P(mU)#(fG)#(mG)(fA)(mG)(fA)(mA)(fG)(mG)(fA)(mU)(fA)(mC)#(fU)#(mC)#(fA)#(mU)#(fU)#(mG)#(fA)	(fG)#(mA)#(fG)(mU)(fA)(mU)(fC)(mC)(fU)(mU)(fC)(mU)(fC)#(mC)#(fA)-TegChol
Apoe_1112	Mouse ApoE	NM_009696	P(mU)#(fA)#(mC)(fA)(mG)(fG)(mA)(fG)(mA)(fA)(mG)(fG)(mA)#(fU)#(mA)#(fC)#(mU)#(fC)#(mA)#(fU)	(fU)#(mA)#(fU)(mC)(fC)(mU)(fU)(mC)(fU)(mC)(fC)(mU)(fG)#(mU)#(fA)-TegChol
Apoe_1115	Mouse ApoE	NM_009696	P(mU)#(fA)#(mG)(fG)(mA)(fC)(mA)(fG)(mG)(fA)(mG)(fA)(mA)#(fG)#(mG)#(fA)#(mU)#(fA)#(mC)#(fU)	(fC)#(mC)#(fU)(mU)(fC)(mU)(fC)(mC)(fU)(mG)(fU)(mC)(fC)#(mU)#(fA)-TegChol
Apoe_1119	Mouse ApoE	NM_009696	P(mU)#(fU)#(mU)(fG)(mC)(fA)(mG)(fG)(mA)(fC)(mA)(fG)(mG)#(fA)#(mG)#(fA)#(mA)#(fG)#(mG)#(fA)	(fC)#(mU)#(fC)(mC)(fU)(mG)(fU)(mC)(fC)(mU)(fG)(mC)(fA)#(mA)#(fA)-TegChol
Apoe_1121	Mouse ApoE	NM_009696	P(mU)#(fU)#(mG)(fU)(mU)(fG)(mC)(fA)(mG)(fG)(mA)(fC)(mA)#(fG)#(mG)#(fA)#(mG)#(fA)#(mA)#(fG)	(fC)#(mC)#(fU)(mG)(fU)(mC)(fC)(mU)(fG)(mC)(fA)(mA)(fC)#(mA)#(fA)-TegChol
Apoe_1123	Mouse ApoE	NM_009696	P(mU)#(fG)#(mU)(fU)(mG)(fU)(mU)(fG)(mC)(fA)(mG)(fG)(mA)#(fC)#(mA)#(fG)#(mG)#(fA)#(mG)#(fA)	(fU)#(mG)#(fU)(mC)(fC)(mU)(fG)(mC)(fA)(mA)(fC)(mA)(fA)#(mC)#(fA)-TegChol
Apoe_1124	Mouse ApoE	NM_009696	P(mU)#(fU)#(mG)(fU)(mU)(fG)(mU)(fU)(mG)(fC)(mA)(fG)(mG)#(fA)#(mC)#(fA)#(mG)#(fG)#(mA)#(fG)	(fG)#(mU)#(fC)(mC)(fU)(mG)(fC)(mA)(fA)(mC)(fA)(mA)(fC)#(mA)#(fA)-TegChol
Apoe_1126	Mouse ApoE	NM_009696	P(mU)#(fG)#(mA)(fU)(mG)(fU)(mU)(fG)(mU)(fU)(mG)(fC)(mA)#(fG)#(mG)#(fA)#(mC)#(fA)#(mG)#(fG)	(fC)#(mC)#(fU)(mG)(fC)(mA)(fA)(mC)(fA)(mA)(fC)(mA)(fU)#(mC)#(fA)-TegChol
Apoe_1129	Mouse ApoE	NM_009696	P(mU)#(fA)#(mU)(fG)(mG)(fA)(mU)(fG)(mU)(fU)(mG)(fU)(mU)#(fG)#(mC)#(fA)#(mG)#(fG)#(mA)#(fC)	(fG)#(mC)#(fA)(mA)(fC)(mA)(fA)(mC)(fA)(mU)(fC)(mC)(fA)#(mU)#(fA)-TegChol
Apoe_1134	Mouse ApoE	NM_009696	P(mU)#(fU)#(mG)(fG)(mA)(fU)(mA)(fU)(mG)(fG)(mA)(fU)(mG)#(fU)#(mU)#(fG)#(mU)#(fU)#(mG)#(fC)	(fA)#(mA)#(fC)(mA)(fU)(mC)(fC)(mA)(fU)(mA)(fU)(mC)(fC)#(mA)#(fA)-TegChol
Apoe_1141	Mouse ApoE	NM_009696	P(mU)#(fA)#(mC)(fC)(mU)(fG)(mG)(fC)(mU)(fG)(mG)(fA)(mU)#(fA)#(mU)#(fG)#(mG)#(fA)#(mU)#(fG)	(fA)#(mU)#(fA)(mU)(fC)(mC)(fA)(mG)(fC)(mC)(fA)(mG)(fG)#(mU)#(fA)-TegChol
Apoe_1146	Mouse ApoE	NM_009696	P(mU)#(fG)#(mG)(fG)(mC)(fC)(mA)(fC)(mC)(fU)(mG)(fG)(mC)#(fU)#(mG)#(fG)#(mA)#(fU)#(mA)#(fU)	(fC)#(mA)#(fG)(mC)(fC)(mA)(fG)(mG)(fU)(mG)(fG)(mC)(fC)#(mC)#(fA)-TegChol
Apoe_1163	Mouse ApoE	NM_009696	P(mU)#(fG)#(mA)(fG)(mA)(fG)(mG)(fU)(mG)(fC)(mU)(fU)(mG)#(fA)#(mG)#(fA)#(mC)#(fA)#(mG)#(fG)	(fC)#(mU)#(fC)(mA)(fA)(mG)(fC)(mA)(fC)(mC)(fU)(mC)(fU)#(mC)#(fA)-TegChol
Apoe_1167	Mouse ApoE	NM_009696	P(mU)#(fG)#(mC)(fC)(mA)(fG)(mA)(fG)(mA)(fG)(mG)(fU)(mG)#(fC)#(mU)#(fU)#(mG)#(fA)#(mG)#(fA)	(fA)#(mG)#(fC)(mA)(fC)(mC)(fU)(mC)(fU)(mC)(fU)(mG)(fG)#(mC)#(fA)-TegChol

Apoe_1188	Mouse ApoE	NM_009696	P(mU)#(fU)#(mU)(fA)(mA)(fG)(mC)(fA)(mA)(fG)(mG)(fG)(mC)#(fC)#(mA)#(fC)#(mC)#(fA)#(mG)#(fA)	(fU)#(mG)#(fG)(mC)(fC)(mC)(fU)(mU)(fG)(mC)(fU)(mU)(fA)#(mA)#(fA)-TegChol
Apoe_1191	Mouse ApoE	NM_009696	P(mU)#(fU)#(mU)(fA)(mU)(fU)(mA)(fA)(mG)(fC)(mA)(fA)(mG)#(fG)#(mG)#(fC)#(mC)#(fA)#(mC)#(fC)	(fC)#(mC)#(fC)(mU)(fU)(mG)(fC)(mU)(fU)(mA)(fA)(mU)(fA)#(mA)#(fA)-TegChol
Apoe_1192	Mouse ApoE	NM_009696	P(mU)#(fU)#(mU)(fU)(mA)(fU)(mU)(fA)(mA)(fG)(mC)(fA)(mA)#(fG)#(mG)#(fG)#(mC)#(fC)#(mA)#(fC)	(fC)#(mC)#(fU)(mU)(fG)(mC)(fU)(mU)(fA)(mA)(fU)(mA)(fA)#(mA)#(fA)-TegChol
Apoe_1196	Mouse ApoE	NM_009696	P(mU)#(fA)#(mA)(fU)(mC)(fU)(mU)(fU)(mA)(fU)(mU)(fA)(mA)#(fG)#(mC)#(fA)#(mA)#(fG)#(mG)#(fG)	(fG)#(mC)#(fU)(mU)(fA)(mA)(fU)(mA)(fA)(mA)(fG)(mA)(fU)#(mU)#(fA)-TegChol
Apoe_1203	Mouse ApoE	NM_009696	P(mU)#(fC)#(mU)(fC)(mG)(fG)(mA)(fG)(mA)(fA)(mU)(fC)(mU)#(fU)#(mU)#(fA)#(mU)#(fU)#(mA)#(fA)	(fA)#(mA)#(fA)(mG)(fA)(mU)(fU)(mC)(fU)(mC)(fC)(mG)(fA)#(mG)#(fA)-TegChol
Apoe_1209	Mouse ApoE	NM_009696	P(mU)#(fA)#(mA)(fU)(mG)(fU)(mG)(fC)(mU)(fC)(mG)(fG)(mA)#(fG)#(mA)#(fA)#(mU)#(fC)#(mU)#(fU)	(fU)#(mC)#(fU)(mC)(fC)(mG)(fA)(mG)(fC)(mA)(fC)(mA)(fU)#(mU)#(fA)-TegChol
Apoe_1213	Mouse ApoE	NM_009696	P(mU)#(fU)#(mC)(fA)(mG)(fA)(mA)(fU)(mG)(fU)(mG)(fC)(mU)#(fC)#(mG)#(fG)#(mA)#(fG)#(mA)#(fA)	(fC)#(mG)#(fA)(mG)(fC)(mA)(fC)(mA)(fU)(mU)(fC)(mU)(fG)#(mA)#(fA)-TegChol
Apoe_1213	Mouse ApoE	NM_009696	P(mU)#(fU)#(mC)(fA)(mG)(fA)(mA)(fU)(mG)(fU)(mG)(fC)(mU)#(fC)#(mG)#(fG)#(mA)#(fG)#(mA)#(fA)	(fC)#(mG)#(fA)(mG)(fC)(mA)(fC)(mA)(fU)(mU)(fC)(mU)(fG)#(mA)#(fA)-TegChol
Apoe_1217	Mouse ApoE	NM_009696	P(mU)#(fA)#(mG)(fA)(mC)(fU)(mC)(fA)(mG)(fA)(mA)(fU)(mG)#(fU)#(mG)#(fC)#(mU)#(fC)#(mG)#(fG)	(fC)#(mA)#(fC)(mA)(fU)(mU)(fC)(mU)(fG)(mA)(fG)(mU)(fC)#(mU)#(fA)-TegChol
Apoe_1219	Mouse ApoE	NM_009696	P(mU)#(fA)#(mG)(fA)(mG)(fA)(mC)(fU)(mC)(fA)(mG)(fA)(mA)#(fU)#(mG)#(fU)#(mG)#(fC)#(mU)#(fC)	(fC)#(mA)#(fU)(mU)(fC)(mU)(fG)(mA)(fG)(mU)(fC)(mU)(fC)#(mU)#(fA)-TegChol
Apoe_1223	Mouse ApoE	NM_009696	P(mU)#(fU)#(mC)(fA)(mC)(fA)(mG)(fA)(mG)(fA)(mC)(fU)(mC)#(fA)#(mG)#(fA)#(mA)#(fU)#(mG)#(fU)	(fC)#(mU)#(fG)(mA)(fG)(mU)(fC)(mU)(fC)(mU)(fG)(mU)(fG)#(mA)#(fA)-TegChol
Apoe_1227	Mouse ApoE	NM_009696	P(mU)#(fU)#(mC)(fA)(mC)(fU)(mC)(fA)(mC)(fA)(mG)(fA)(mG)#(fA)#(mC)#(fU)#(mC)#(fA)#(mG)#(fA)	(fG)#(mU)#(fC)(mU)(fC)(mU)(fG)(mU)(fG)(mA)(fG)(mU)(fG)#(mA)#(fA)-TegChol
Apoe_1230	Mouse ApoE	NM_009696	P(mU)#(fG)#(mA)(fA)(mU)(fC)(mA)(fC)(mU)(fC)(mA)(fC)(mA)#(fG)#(mA)#(fG)#(mA)#(fC)#(mU)#(fC)	(fU)#(mC)#(fU)(mG)(fU)(mG)(fA)(mG)(fU)(mG)(fA)(mU)(fU)#(mC)#(fA)-TegChol
Apoe_1233	Mouse ApoE	NM_009696	P(mU)#(fU)#(mU)(fG)(mG)(fA)(mA)(fU)(mC)(fA)(mC)(fU)(mC)#(fA)#(mC)#(fA)#(mG)#(fA)#(mG)#(fA)	(fG)#(mU)#(fG)(mA)(fG)(mU)(fG)(mA)(fU)(mU)(fC)(mC)(fA)#(mA)#(fA)-TegChol

Detailed sequence and chemical modification patterns, of siRNAs. Chemical modifications are designated as follows, “#” –phosphorothioate bond, “m” – 2’-O-Methyl, “f” – 2’-Fluoro, “P” – 5’ Phosphate.

Table 2: Partially modified sense strand sequences and chemical pattern

Name	Gene	Accession Number	Sense strand
Apoe_156	Mouse ApoE	NM_009696	(fA)#(mU)#(fC)(rA)(rA)(rU)(fG)(mA)(fG)(mU)(fA)(mU)(fC)#(mC)#(fA)-TegChol
Apoe_416	Mouse ApoE	NM_009696	(fA)#(mA)#(fU)(rG)(rA)(rG)(fU)(mA)(fU)(mC)(fC)(mU)(fU)#(mC)#(fA)-TegChol
Apoe_636	Mouse ApoE	NM_009696	(fG)#(mA)#(fG)(rU)(rA)(rU)(fC)(mC)(fU)(mU)(fC)(mU)(fC)#(mC)#(fA)-TegChol
Apoe_1004	Mouse ApoE	NM_009696	(fU)#(mA)#(fU)(rC)(rC)(rU)(fU)(mC)(fU)(mC)(fC)(mU)(fG)#(mU)#(fA)-TegChol
Apoe_1032	Mouse ApoE	NM_009696	(fC)#(mC)#(fU)(rU)(rC)(rU)(fC)(mC)(fU)(mG)(fU)(mC)(fC)#(mU)#(fA)-TegChol
Apoe_1103	Mouse ApoE	NM_009696	(fC)#(mC)#(fU)(rG)(rU)(rC)(fC)(mU)(fG)(mC)(fA)(mA)(fC)#(mA)#(fA)-TegChol
Apoe_1106	Mouse ApoE	NM_009696	(fG)#(mU)#(fC)(rC)(rU)(rG)(fC)(mA)(fA)(mC)(fA)(mA)(fC)#(mA)#(fA)-TegChol
Apoe_1109	Mouse ApoE	NM_009696	(fG)#(mC)#(fA)(rA)(rC)(rA)(fA)(mC)(fA)(mU)(fC)(mC)(fA)#(mU)#(fA)-TegChol
Apoe_1112	Mouse ApoE	NM_009696	(fA)#(mA)#(fC)(rA)(rU)(rC)(fC)(mA)(fU)(mA)(fU)(mC)(fC)#(mA)#(fA)-TegChol
Apoe_1115	Mouse ApoE	NM_009696	(fA)#(mU)#(fA)(rU)(rC)(rC)(fA)(mG)(fC)(mC)(fA)(mG)(fG)#(mU)#(fA)-TegChol
Apoe_1119	Mouse ApoE	NM_009696	(fC)#(mA)#(fG)(rC)(rC)(rA)(fG)(mG)(fU)(mG)(fG)(mC)(fC)#(mC)#(fA)-TegChol
Apoe_1121	Mouse ApoE	NM_009696	(fC)#(mU)#(fC)(rA)(rA)(rG)(fC)(mA)(fC)(mC)(fU)(mC)(fU)#(mC)#(fA)-TegChol
Apoe_1123	Mouse ApoE	NM_009696	(fA)#(mG)#(fC)(rA)(rC)(rC)(fU)(mC)(fU)(mC)(fU)(mG)(fG)#(mC)#(fA)-TegChol
Apoe_1124	Mouse ApoE	NM_009696	(fU)#(mG)#(fG)(rC)(rC)(rC)(fU)(mU)(fG)(mC)(fU)(mU)(fA)#(mA)#(fA)-TegChol
Apoe_1126	Mouse ApoE	NM_009696	(fC)#(mC)#(fU)(rU)(rG)(rC)(fU)(mU)(fA)(mA)(fU)(mA)(fA)#(mA)#(fA)-TegChol
Apoe_1129	Mouse ApoE	NM_009696	(fG)#(mC)#(fU)(rU)(rA)(rA)(fU)(mA)(fA)(mA)(fG)(mA)(fU)#(mU)#(fA)-TegChol
Apoe_1134	Mouse ApoE	NM_009696	(fA)#(mA)#(fA)(rG)(rA)(rU)(fU)(mC)(fU)(mC)(fC)(mG)(fA)#(mG)#(fA)-TegChol
Apoe_1141	Mouse ApoE	NM_009696	(fU)#(mC)#(fU)(rC)(rC)(rG)(fA)(mG)(fC)(mA)(fC)(mA)(fU)#(mU)#(fA)-TegChol
Apoe_1146	Mouse ApoE	NM_009696	(fC)#(mG)#(fA)(rG)(rC)(rA)(fC)(mA)(fU)(mU)(fC)(mU)(fG)#(mA)#(fA)-TegChol
Apoe_1163	Mouse ApoE	NM_009696	(fC)#(mA)#(fU)(rU)(rC)(rU)(fG)(mA)(fG)(mU)(fC)(mU)(fC)#(mU)#(fA)-TegChol
Apoe_1167	Mouse ApoE	NM_009696	(fC)#(mU)#(fG)(rA)(rG)(rU)(fC)(mU)(fC)(mU)(fG)(mU)(fG)#(mA)#(fA)-TegChol
Apoe_1188	Mouse ApoE	NM_009696	(fG)#(mU)#(fC)(rU)(rC)(rU)(fG)(mU)(fG)(mA)(fG)(mU)(fG)#(mA)#(fA)-TegChol
Apoe_1191	Mouse ApoE	NM_009696	(fU)#(mC)#(fU)(rG)(rU)(rG)(fA)(mG)(fU)(mG)(fA)(mU)(fU)#(mC)#(fA)-TegChol
Apoe_1192	Mouse ApoE	NM_009696	(fG)#(mU)#(fG)(rA)(rG)(rU)(fG)(mA)(fU)(mU)(fC)(mC)(fA)#(mA)#(fA)-TegChol
Apoe_1196	Mouse ApoE	NM_009696	(fA)#(mU)#(fC)(rA)(rA)(rU)(fG)(mA)(fG)(mU)(fA)(mU)(fC)#(mC)#(fA)-TegChol
Apoe_1203	Mouse ApoE	NM_009696	(fA)#(mA)#(fU)(rG)(rA)(rG)(fU)(mA)(fU)(mC)(fC)(mU)(fU)#(mC)#(fA)-TegChol
Apoe_1209	Mouse ApoE	NM_009696	(fG)#(mA)#(fG)(rU)(rA)(rU)(fC)(mC)(fU)(mU)(fC)(mU)(fC)#(mC)#(fA)-TegChol
Apoe_1213	Mouse ApoE	NM_009696	(fU)#(mA)#(fU)(rC)(rC)(rU)(fU)(mC)(fU)(mC)(fC)(mU)(fG)#(mU)#(fA)-TegChol
Apoe_1213	Mouse ApoE	NM_009696	(fC)#(mC)#(fU)(rU)(rC)(rU)(fC)(mC)(fU)(mG)(fU)(mC)(fC)#(mU)#(fA)-TegChol
Apoe_1217	Mouse ApoE	NM_009696	(fC)#(mC)#(fU)(rG)(rU)(rC)(fC)(mU)(fG)(mC)(fA)(mA)(fC)#(mA)#(fA)-TegChol
Apoe_1219	Mouse ApoE	NM_009696	(fG)#(mU)#(fC)(rC)(rU)(rG)(fC)(mA)(fA)(mC)(fA)(mA)(fC)#(mA)#(fA)-TegChol
Apoe_1223	Mouse ApoE	NM_009696	(fG)#(mC)#(fA)(rA)(rC)(rA)(fA)(mC)(fA)(mU)(fC)(mC)(fA)#(mU)#(fA)-TegChol
Apoe_1227	Mouse ApoE	NM_009696	(fA)#(mA)#(fC)(rA)(rU)(rC)(fC)(mA)(fU)(mA)(fU)(mC)(fC)#(mA)#(fA)-TegChol
Apoe_1230	Mouse ApoE	NM_009696	(fA)#(mU)#(fA)(rU)(rC)(rC)(fA)(mG)(fC)(mC)(fA)(mG)(fG)#(mU)#(fA)-TegChol
Apoe_1233	Mouse ApoE	NM_009696	(fC)#(mA)#(fG)(rC)(rC)(rA)(fG)(mG)(fU)(mG)(fG)(mC)(fC)#(mC)#(fA)-TegChol

Detailed sequence and chemical modification patterns, of siRNAs. Chemical modifications are designated as follows, “#” –phosphorothioate bond, “m” – 2’-O-Methyl, “f” – 2’-Fluoro, “P” – 5’ Phosphate.

Table 3: Key Resources

Reagent	Source	Identifier
**QuantiGene Probeset**		
Mouse ApoE	Thermofisher	SB-13611
Mouse PPIB	Thermofisher	SB-10002
**Antibodies**		
Rabbit polyclonal anti-ApoE	Abcam	183597
Rabbit polyclonal anti-beta-actin	Cell Signaling Technologies	4970
Anti-NeuN	Millipore	MAB37
Anti-GFAP	Abcam	5441
**RNAscope Probe sets**		
ApoE	ACDBio	313271
HPRT	ACDBio	312951
**Commerical Assays**		
QuantiGene 2.0	Thermofisher	QS0011
RNAscope Fluorescent Multiplex Kit	ACDBio	320850
**Deposited Data**		
N2A cells	GSE45119	
Astrocytes	GSE52564	
**Primers**	Sequence	
ApoE Forward	GCTCAGACCCTGGAGGCTAA	
ApoE Reverse	CTGTTCCTCCAGCTCCTTTTTGTA	
